# Purine metabolism enhances neovascularization of type H vessels in the induced membrane technique

**DOI:** 10.1172/jci.insight.200820

**Published:** 2026-07-22

**Authors:** Yung-Heng Hsu, Guan-Lin Lee, Yu-Chih Lin, Mei-Feng Chen, Yuhan Chang, Ying-Yu Wu, Chih-Chien Hu

**Affiliations:** 1 Bone and Joint Research Center and; 2 Department of Orthopedic Surgery, Chang Gung Memorial Hospital, Taoyuan, Taiwan.; 3 College of Medicine, Chang Gung University, Taoyuan, Taiwan.

**Keywords:** Metabolism, Vascular biology, Angiogenesis, Endothelial cells, Metabolomics

## Abstract

The induced membrane technique (IMT) is a 2-stage surgical intervention for critical-sized bone defects (CSBD), yet the metabolic mechanisms driving neovascularization within the induced membrane remain unclear. Here, we combined a rat IMT model, metabolomic profiling, and endothelial assays to delineate the role of purine metabolism in neovascularization of type H vessels. Using a rat IMT model and metabolomic profiling, we identified purine metabolism as the most substantial pathway during the formation of induced membranes, with consistent trends of adenosine, inosine, hypoxanthine, and xanthosine found in both serum and induced membranes. Histological analysis revealed abundant CD31^hi^EMCN^hi^ type H vessels, critical for osteogenesis, within the induced membrane. Inhibition of purine metabolism suppressed the CD31^hi^EMCN^hi^ type H phenotype in human umbilical vein endothelial cells, whereas treatment with inosine, hypoxanthine, or xanthosine promoted endothelial activation and the type H phenotype. Notably, inosine and hypoxanthine displayed parallel changes across consistent systemic (serum) and local alterations (induced membranes), highlighting their potential as serum indicators of induced membrane formation. Collectively, these findings uncover a previously unrecognized metabolic mechanism driving neovascularization of type H vessels in induced membranes and suggest purine metabolites as promising indicators and therapeutic targets for improving IMT outcomes as well as CBSD treatment.

## Abstract 2

Purine metabolism promotes growth of a specialized blood vessel type in induced membranes, offering insight into how large bone defects may heal more effectively.

## Graphical abstract

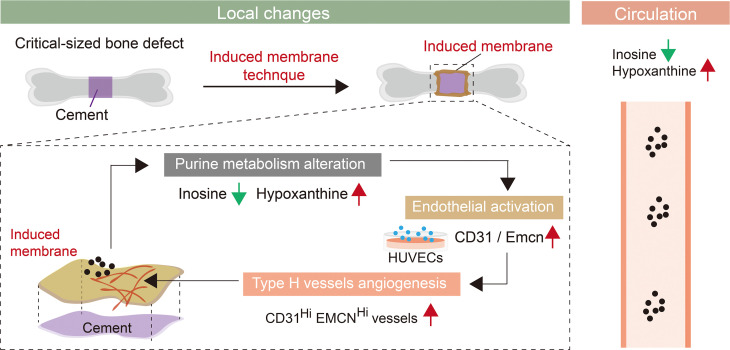

## Introduction

The Masquelet technique, or induced membrane technique (IMT), has emerged as a promising strategy to enhance the healing of critical-sized bone defects (CSBDs). By inducing membrane maturation through a staged process, IMT offers an effective alternative to traditional approaches, including vascularized bone grafting, bone transport, and autologous bone grafting (1–3). IMT was first introduced by Masquelet et al. (4) in 1986 as a 2-stage surgical operation to treat CSBDs of up to 25 cm in length. A CSBD usually results from high-energy trauma, infection, and surgical resection of tumors, and it is defined as the minimal size of bone loss that cannot spontaneously heal within 9 months (1–3). In IMT, stage I involves the implantation of a polymethylmethacrylate (PMMA) cement spacer into the defect site to form induced membranes via a foreign-body immune response (1–4). In stage II, the spacer is carefully removed while maintaining the induced membrane at the defect site, and an autologous bone graft is performed to promote bone healing (1–4). As the most effective surgical intervention for CSBD, IMT has several challenges, including inadequate membrane formation, financial strain, multiple surgeries, and a long recovery time. Thus, the simplification of IMT in a one-stage surgery using artificial materials loaded with osteogenic biomolecules is a highly researched subject. To advance this concept and address residual infections—a common cause of failure in single-stage surgeries—Yu et al. designed a composite artificial membrane enriched with antibiotics and bone morphogenetic protein 2 (BMP2) (5). In a related study, Raina et al. employed a calcium sulfate/hydroxyapatite biomaterial capable of delivering BMP2 and zoledronic acid in a controlled spatiotemporal manner (6). Despite these studies, there is currently no definitive method in orthopedics to predict the formation of induced membranes and simplify the IMT procedure. In IMT, the induced membrane is essential for new bone formation, and elucidating its mechanisms will be instrumental in simplifying the surgical approach. Thus, identifying biomolecules from induced membranes will help overcome the clinical challenges associated with IMT-treated CSBD.

The induced membrane, which functions as a pseudoperiosteum, is a key factor in promoting bone formation in the IMT to treat CSBD (2, 4, 5). Current research suggests that this effect is attributed mainly to the presence of enriched angiogenic factors and activated endothelial cells (7–9), which drive the neovascularization of new capillaries (10, 11). However, the underlying mechanisms remain poorly understood. Type H vessels are a specialized subtype of bone capillaries characterized by a high expression of CD31 and endomucin (CD31^hi^ EMCN^hi^) (12, 13). Despite this, whether type H vessels are present in the induced membrane to regulate new bone formation remains unclear.

Morphologically, type H vessels exhibit a columnar structure and are primarily located in the metaphysis as well as in both the periosteum and endosteum of the diaphysis (14, 15). These vessels have been shown to contribute critically to fracture healing and osteogenesis (14, 16). Previous studies have reported that the microenvironment surrounding type H vessels is enriched with angiogenic factors and osteogenic cells, further supporting their role in bone regeneration (12, 13). A study by Wang et al. further demonstrated that a decline in type H vessel formation is associated with bone loss in patients with hip fractures, suggesting that these vessels could serve as promising biomarkers for bone formation (17). Based on the available evidence, we hypothesized that type H vessels are critical in induced membrane–mediated osteogenesis and facilitating CSBD healing. Similar to other vascular networks, endothelial cell activation is essential for cell growth and migration during angiogenesis of type H vessels (14, 16). However, whether the activated endothelial cells in the induced membrane exhibit the CD31^hi^EMCN^hi^ phenotype remains unclear. Given the potential significance of these vessels in IMT-mediated bone regeneration, identifying biomolecules and regulatory mechanisms associated with type H vessel formation within the induced membrane is crucial for advancing our understanding and optimizing IMT outcomes.

Utilizing a high-throughput metabolomics approach to comprehensively analyze biological samples has enabled the identification of regulated metabolites and the characterization of metabolic phenotypes in various diseases, including cancer and kidney fibrosis (18, 19). It has been used in bone diseases to provide metabolic insights into osteoporosis, bone mineral density, and inflammatory bone diseases (20–23). In our previous report, metabolome analysis revealed that purine metabolism is highly regulated and that inosine enhances fracture healing by promoting the formation of type H vessels (24). However, metabolomics has never been used to analyze metabolic regulation in IMT. Thus, it is crucial to explore the mechanism of induced membranes from a alternative perspective, particularly the metabolic regulation of angiogenesis, to facilitate CSBD healing by uncovering influencing metabolites. Additionally, it holds potential for simplifying the IMT, thereby reducing clinical challenges in the treatment of CSBD.

In this study, a defect size of 3 mm was created in the femurs of Sprague Dawley (SD) rats to establish a CSBD model for metabolomic analysis using the Xevo G2-XS QT Quadrupole Time-of-Flight Mass Spectrometry in a well-equipped Clinical Metabolomics Core Lab of our institute. Metabolomics data provided metabolic insights into the serum and tissues of these rats; comparisons between systemic and local alterations enabled the determination of their potential as convenient indicators for induced membrane formation. Furthermore, our in vitro data from human umbilical vein endothelial cells (HUVEC), a well-known in vitro model for studying angiogenesis, corroborated the role of the identified metabolites in regulating type H markers and the angiogenic capacity of type H vessels. This study aimed to identify potential metabolites from induced membranes using a metabolomics approach to provide a potential strategy for the improvement of IMT in orthopedics.

## Results

### Metabolic alteration of purine metabolism in rats with induced membrane technique.

**Figure 1:**
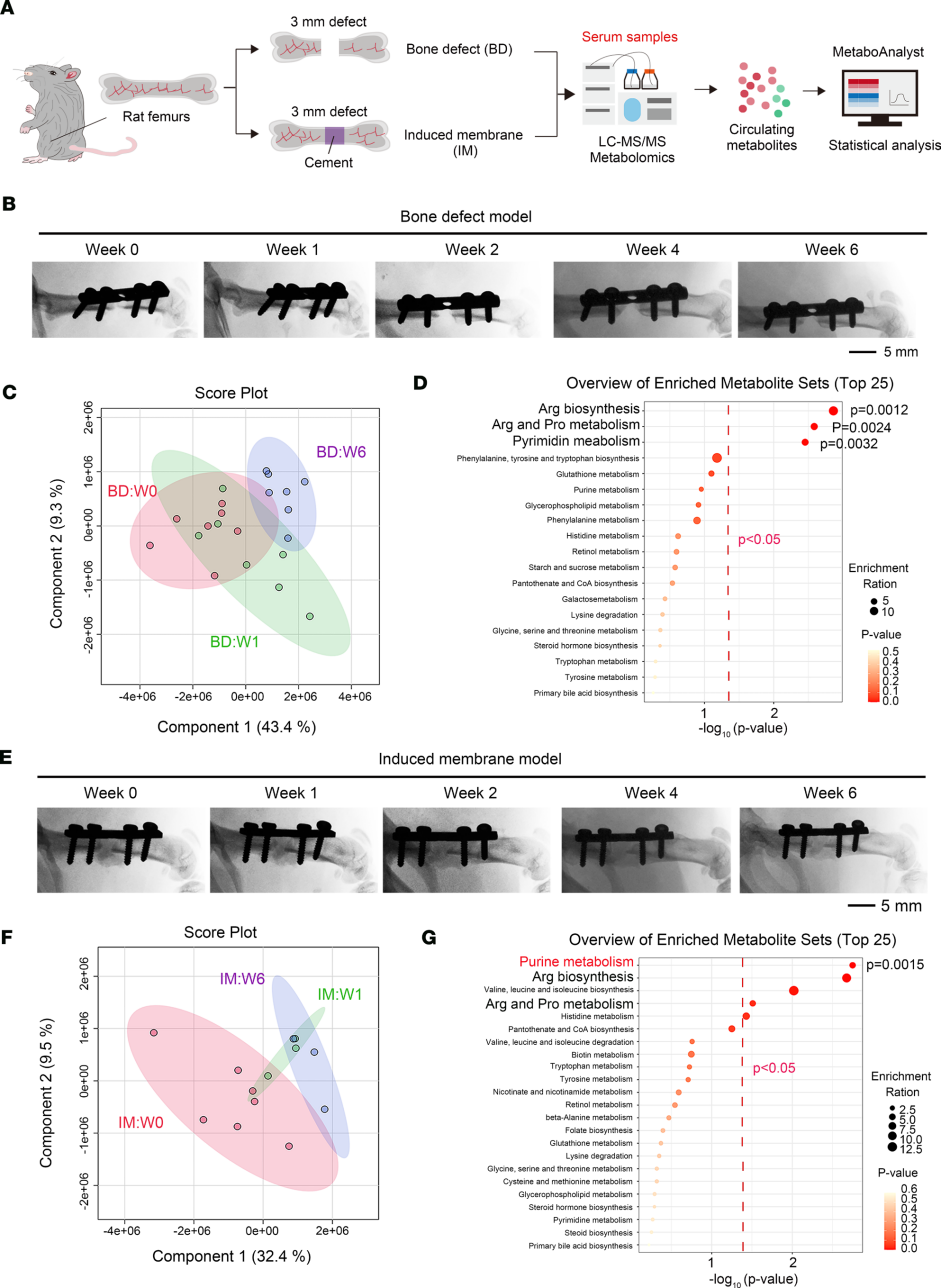
Metabolomic analysis of serum samples from rats undergoing the induced membrane technique. (**A**) Untargeted metabolomic profiling was performed on serum collected from rats subjected to either bone defect (BD) or induced membrane technique (IMT) procedures. Metabolites were considered significant if they met all 3 criteria: *P* < 0.05, FDR < 0.2, and Fold > 1.5. (**B**) In the BD model, 2-dimensional micro-CT scans were performed weekly to monitor bone stability at the 3.0 mm femoral defect site (*n* = 7). (**C**) Serum metabolite patterns were visualized through partial least squares discriminant analysis (PLS-DA) using samples from week 0 (BD:W0, *n* = 7, 3 rats were excluded due to hemolysis), week 1 (BD:W1, *n* = 7), and week 6 (BD:W6, *n* = 7). Each point represents an individual rat. (**D**) Metabolic pathway enrichment for BD rat serum was assessed using MetaboAnalyst (Hypergeometric Test). Pathways were prioritized based on significance level, represented as –log_10_(*P* value), with those to the right of the red threshold line considered statistically relevant. (**E**) For IMT-treated rats, weekly 2D micro-CT images were acquired to track femoral changes following implantation and membrane induction (*n* = 6). (**F**) PLS-DA was also applied to serum samples from IMT rats at 3 time points: week 0 (IM:W0, shared with **C**), week 1 (IM:W1, *n* = 3), and week 6 (IM:W6, *n* = 3), with each dot representing one rat. (**G**) Metabolomics pathway enrichment for IMT serum samples was performed using MetaboAnalyst (hypergeometric test), highlighting altered pathways over time. Only pathways to the right of the red dashed significance threshold line were retained. Week 0 represented in this figure should be 10 rats, from which 3 were excluded due to hemolysis causing incorrect results of metabolomics analysis. Arg, arginine; Pro, proline; Phe, phenylalanine; Tyr, tyrosine; Trp, tryptophan.

Exploring the molecular mechanisms of IMT may provide an effective approach or intervention for CSBD; however, these mechanisms have not yet been clarified by metabolomics. Thus, a CSBD size of 3 mm was introduced into the femurs of rats, and fixed using bone screws (1.5 × 7 mm) together with bone plates (1.0 mm thick). To induce the formation of the induced membrane (IM), PMMA cement was placed into the defect sites for 6 weeks in IMT rats, whereas the defect site remained empty as the bone defect (BD) group (Figure 1A). Animals showed no apparent motor impairment and feeding difficulties after surgery. Serum samples were collected for untargeted metabolome analysis using ultra-performance liquid chromatography-tandem mass spectrometry (UPLC-MS/MS). Subsequently, the serum metabolome analysis identified metabolites in both negative and positive ion modes using MS-DIAL. MetaboAnalyst (https://www.metaboanalyst.ca/MetaboAnalyst/home.xhtml) was then used to process these identified targets through statistical analysis (week 0, week 1, and week 6; *P* value < 0.05, FDR < 0.2 with 1.5 × differential fold change) and subsequent pathway analysis (Figure 1A). The stability of the defects was examined weekly by micro-CT scanning, and the 6th week image indicated the maintenance of defects that mimic the CSBD condition in the BD group (Figure 1B). Partial least squares–discriminant analysis (PLS-DA) results from MetaboAnalyst showed that the 6th week BD rats could be discriminated from those at week 0 and week 1 (Figure 1C). Furthermore, pathway analysis revealed that arginine (Arg) biosynthesis, Arg and proline (Pro) metabolism, and pyrimidine metabolism were the top 3 pathways affected in BD rats (Figure 1D). Consistent with BD rats, IMT rats at week 6 could also be discriminated, as indicated by PLSDA analysis (Figure 1, A and E). Notably, pathway analysis revealed that purine metabolism, which showed no significant difference in the BD group, emerged as the most statistically significant pathway at week 6 in the IMT group (Figure 1, F and G). At both weeks 1 and 6, between-group metabolomic comparisons between IMT and BD were largely dominated by the critical-sized injury response, and the pathway signatures were similar to those observed in the time-course analysis of BD rats (Supplemental Figure 1; supplemental material available online with this article; https://doi.org/10.1172/jci.insight.200820DS1). Therefore, these cross-sectional comparisons were not interpreted as IMT-specific features, and we focused our main analyses on longitudinal and pathway-level dynamics. Compared with other significant pathways, such as arginine biosynthesis and arginine and proline metabolism, purine metabolism showed the greatest degree of alteration. This pronounced change suggests that purine metabolism is not only the most promising metabolic pathway but also a potential mechanistic target of PMMA-mediated membranes in IMT rats. These findings highlight the possibility that purine metabolism plays a critical role in regulating the biological response of the induced membrane, thereby this pathway was selected in this study to address molecular mechanisms of induced membrane.

### Correlation of purine metabolites with the progression of induced membrane formation.

**Figure 2:**
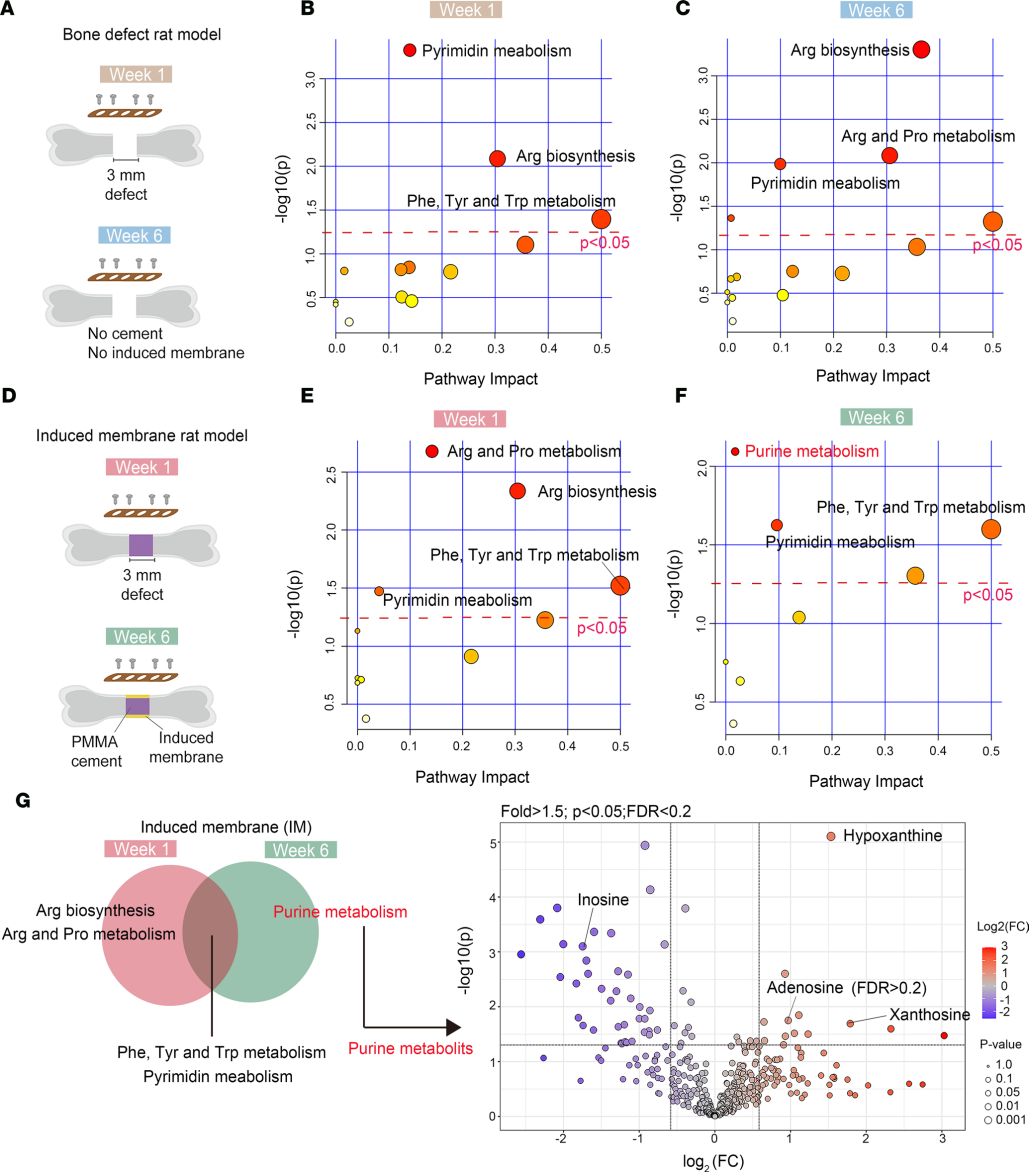
Purine metabolism is actively regulated during the first stage of induced membrane technique. Serum samples were collected from both bone defect (BD) and induced membrane technique (IMT) rats at weeks 0, 1, and 6. To characterize early and late metabolic alterations, comparisons between week 0 and week 1 were defined as the early stage (week 1), while those between week 0 and week 6 were defined as the late stage (week 6). Data shown in Figure 2 were same cohort of Figure 1. (**A**) Schematic of the experimental setup for BD rats. (**B** and **C**) Metabolic pathway enrichment analyses of BD rat serum profiles during the early (**B**, week 0 versus week 1) and late (**C**, week 0 versus week 6) phases. Analyses were performed using MetaboAnalyst (hypergeometric test), which ranked enriched pathways based on adjusted *P* values (color gradient from white to red on the *y* axis) and pathway impact scores (*x* axis), with circle size indicating the number of identified metabolites relative to total pathway components. (**D**) Diagram showing the IMT rat experimental model. (**E** and **F**) Enriched pathway in the IMT group at the early (**E**, week 0 versus week 1) and late (**F**, week 0 versus week 6) phases. Analyses were performed using MetaboAnalyst (hypergeometric test), which ranked enriched pathways based on adjusted *P* values (color gradient from white to red on the *y* axis) and pathway impact scores (*x* axis), with circle size indicating the number of identified metabolites relative to total pathway components. (**G**) The volcano plot (Student’s *t* test) of purine metabolism at the later stage (week 6). Pathways were considered significant if they met the following criteria: *P* < 0.05. Metabolites were considered significant if they met the following criteria: *P* < 0.05, FDR < 0.2, and Fold > 1.5. Arg, arginine; Pro, proline; Phe, phenylalanine; Tyr, tyrosine; Trp, tryptophan.

**Figure 3:**
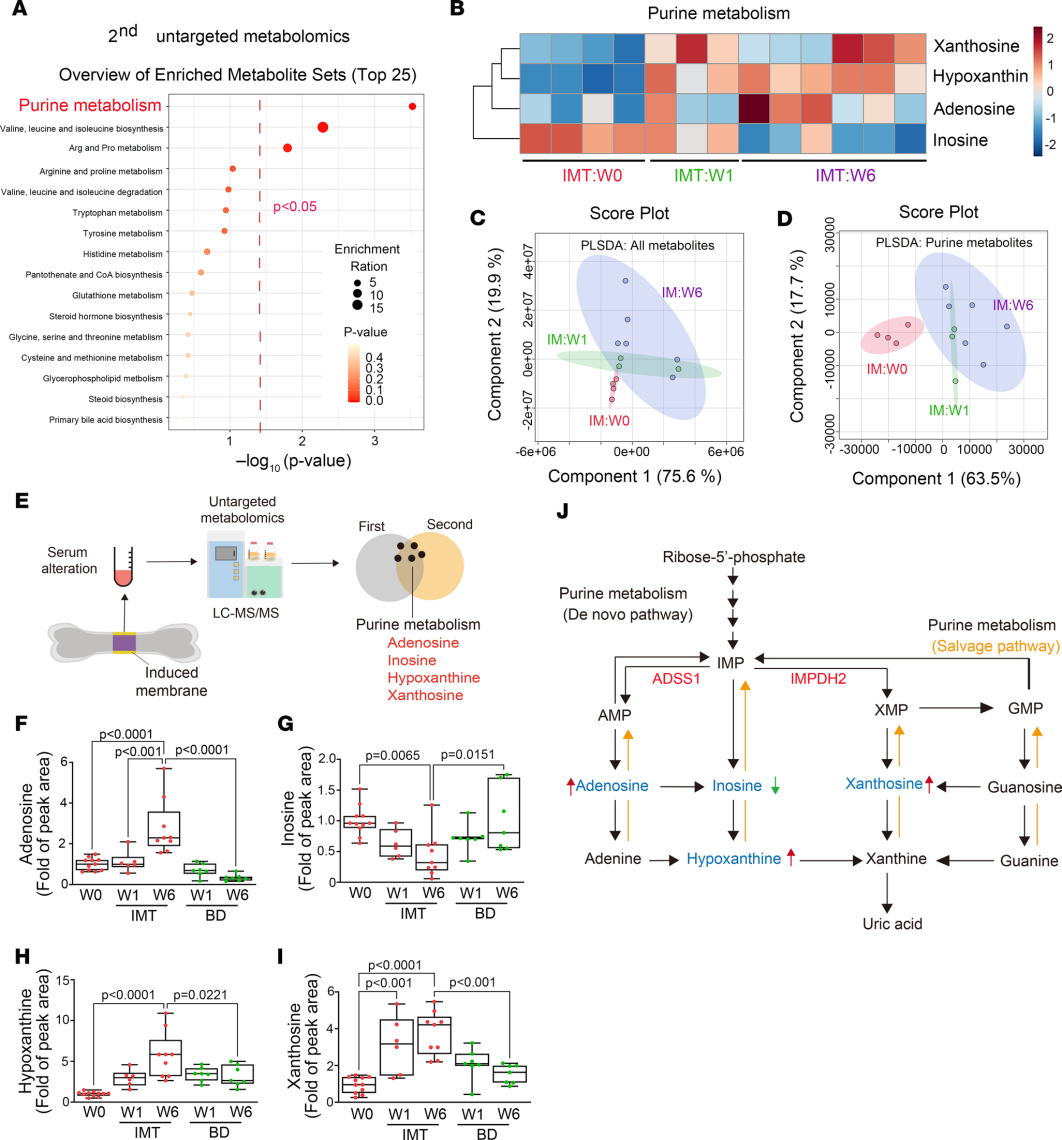
Purine metabolites have the potential to be the indicators of induced membranes. (**A**–**D**) A second independent cohort of induced membrane technique (IMT) rats was analyzed to validate the initial findings for serum metabolomic profiling. Samples were collected at weeks 0 (IMT: W0, *n* = 4; 2 samples excluded due to hemolysis), 1 (IMT: W1, *n* = 3; 3 samples excluded due to hemolysis), and 6 (IMT: W6, *n* = 6). (**A**–**C**) Validation included updated pathway enrichment (**A**, Hypergeometric Test), heatmap visualization of purine-related metabolites identified across both datasets (**B**), and PLS-DA based on the full metabolite dataset (**C**) as well as purine metabolites alone (**D**). Metabolites were considered significant if they met the following criteria: *P* < 0.05, FDR < 0.2, and Fold > 1.5. (**E**) Reproducible identification of purine metabolites from 2 independent analysis of rat serum. (**F**–**I**) Serum profiles of significant purine metabolites—adenosine (**F**), inosine (**G**), hypoxanthine (**H**), and xanthosine (**I**)—identified in both metabolomics datasets. Figure 1 dataset: IMT (week 0, *n* = 7; week 1, *n* = 3; week 6, *n* = 3) and BD rats (week 0, *n* = 7; week 1, *n* = 7; week 6, *n* = 7). Figure 2 dataset: IMT rats (week 0, *n* = 4; week 1, *n* = 3; week 6, *n* = 6). (**J**) Summary of purine metabolism. Box plots represent the median (line), interquartile range (box), and minimum-to-maximum values (whiskers). *P* < 0.05 was considered statistically significant. Blue words (red arrow, upregulation; green arrow, downregulation) indicated identified metabolites in our study. Arg, arginine; BD, bone defect; IM, induced membrane; PLSDA, partial least squares discriminant analysis; Pro, proline.

Since the maturation of the induced membrane is reached at approximately 6 weeks after cement implantation (10), the comparison of pathway analysis between the earlier (week 1) and later (week 6) stages were next performed and compared with the week 0 group in both the BD and IMT groups. The data demonstrated no significant differences in the BD rats between the earlier and later stages (Figure 2, A–C). However, purine metabolism was highly regulated in rats with IMT at the later (week 6) stage (Figure 2, D–F), rather than the earlier stage. The volcano plot by MetaboAnalyst further revealed that downregulation of inosine and upregulation of adenosine, xanthosine, and hypoxanthine were found in serum of IMT rats at week 6 (Figure 2G). To confirm the first metabolomic findings (Figures 1 and 2), fresh serum samples were obtained from a newly prepared group of rats undergoing IMT surgery, followed by a second untargeted metabolomic analysis. Consistent with the first set of findings, the second analysis demonstrated that purine metabolism was still the most prominently altered pathway, with consistent trends observed for adenosine, inosine, hypoxanthine, and xanthosine among the key metabolites (Figure 3, A and B). To next verify the importance of these 4 metabolites, we compared the PLS-DA results obtained from all identified metabolites with those derived solely from the 4 purine metabolites — adenosine, inosine, hypoxanthine, and xanthosine. In line with the PLSDA results clustered by all identified metabolites (Figure 3C), the analysis of the 4 purine metabolites (Figure 3D) indicated a more substantial difference and clustering from the week 0 group in the IMT. Across both metabolomic analyses, purine metabolism was consistently identified as highly regulated, with adenosine, inosine, hypoxanthine, and xanthosine showing reproducible alterations (Figure 3E). By merged results of 2 independent metabolomics analysis, these reverse trends of purine metabolites were further confirmed with normalized data. Consistently, adenosine, hypoxanthine, and xanthosine were upregulated, whereas inosine was downregulated in IMT rats compared with the BD group (Figure 3, F–I). More importantly, these 4 metabolites are highly interconnected within purine metabolism, suggesting that they may jointly regulate related processes to promote induced membrane formation (Figure 3J). Taken together, these findings highlight the strong potential of these metabolites to serve as indicators of IMT.

**Figure 4:**
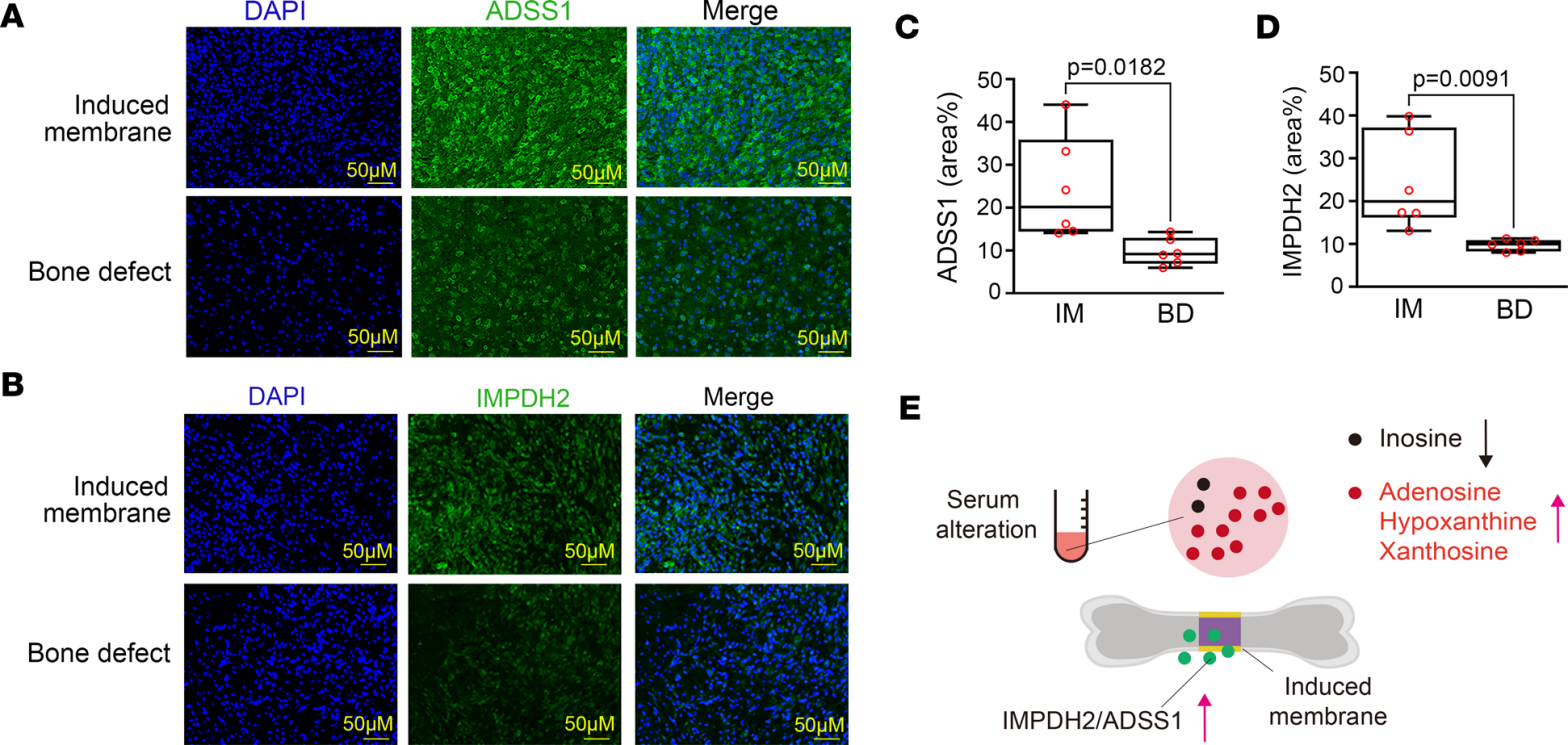
Purinergic enzymes are upregulated in the induced membrane. (**A**–**D**) Representative immunohistochemical images showing expression of ADSS1 (**A**) and IMPDH2 (**B**) in the induced membrane. Quantification of ADSS1 (**C**) and IMPDH2 (**D**) staining using ImageJ. Each dot represents one animal (IM, *n* = 6; BD, *n* = 6). Data are presented as area fraction and normalized to DAPI areas. Box plots represent the median (line), interquartile range (box), and minimum to maximum values (whiskers). *P* < 0.05 was considered statistically significant. (**E**) Summary of Figure 3 showing alterations in serum and induced membrane at week 6 in IMT rats. Scale bars: 50 μm. ADSS1, adenylosuccinate synthase 1; IMPDH2, inosine monophosphate dehydrogenase 2; BD, bone defect; IM, induced membrane.

In the induced membrane, adenylosuccinate synthase 1 (ADSS1) and inosine monophosphate dehydrogenase 2 (IMPDH2), 2 key enzymes in the de novo purine biosynthesis pathway, were expressed at higher levels than in the defective tissues of the BD group (Supplemental Figure 2 and Figure 4, A–D). This finding suggests that the activity of purine metabolism is elevated in the induced membrane, in parallel with circulating changes in adenosine, inosine, hypoxanthine, and xanthosine. These data collectively corroborate that purine metabolism and purine metabolites are potential indicators of the membrane formation induced by IMT (Figure 4E).

### Type H vessel is found in the induced membrane from a CSBD model of rats.

**Figure 5:**
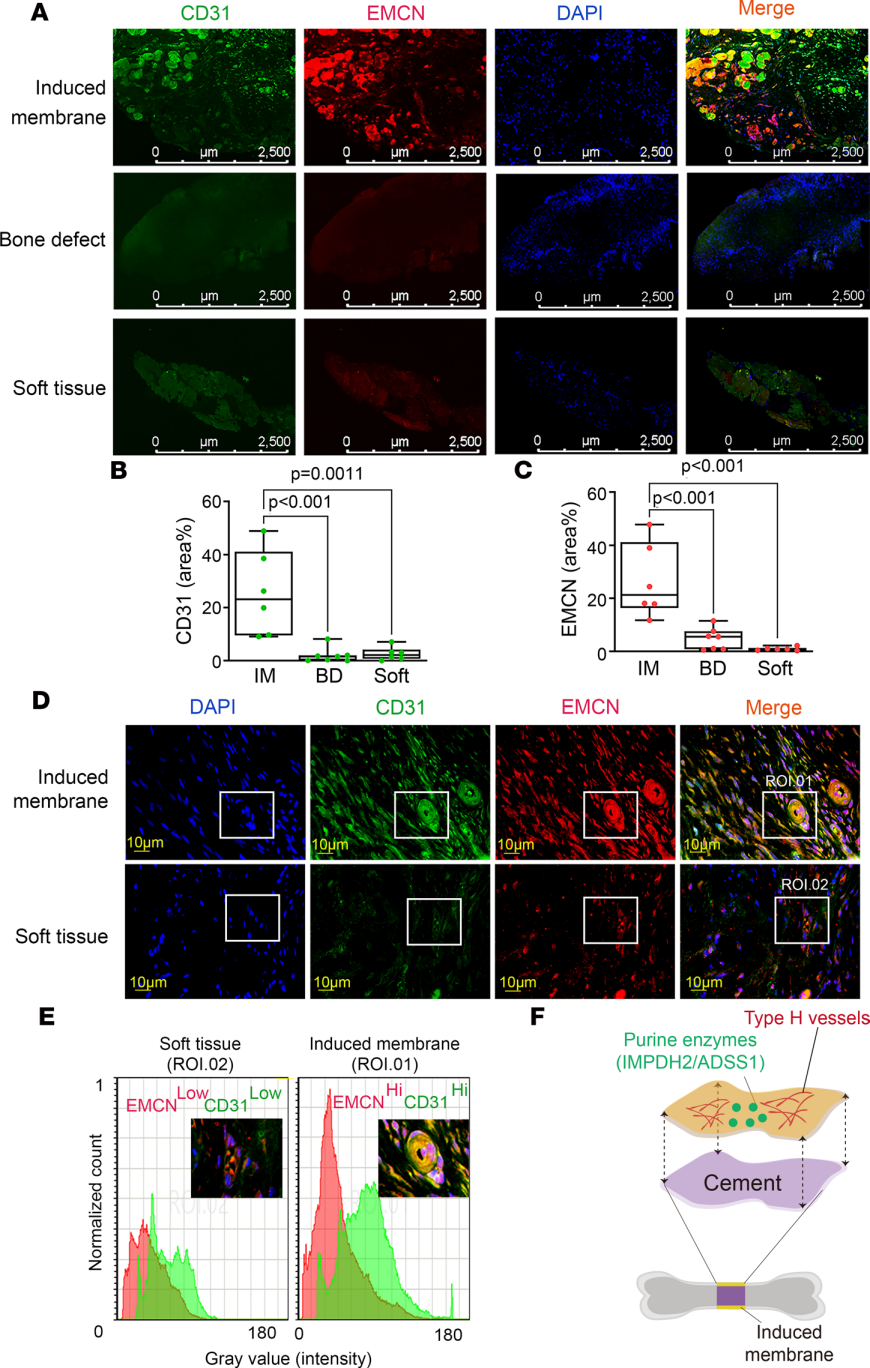
Phenotype of type H vessels is identified in the induced membrane. (**A**) Representative immunohistochemical images showing type H vessels coexpressing CD31 (green) and EMCN (red), with merged orange-yellow signals, in induced membranes (IMT, *n* = 6), bone defect sites (BD, *n* = 7), and soft tissues adjacent to uninjured bone (*n* = 6; randomly selected from IMT and BD rats). (**B** and **C**) Quantification of CD31 and EMCN signals using ImageJ. Each dot represents 1 rat. Data are presented as area fraction and normalized to DAPI areas. (**D**) Immunofluorescence images showing merged staining of DAPI (blue), CD31 (green), and EMCN (red) in induced membranes and soft tissues (*n* = 6 per group). The white box in the upper panels (ROI.01) indicates a type H vessel. (**E**) Quantification of CD31^hi^EMCN^hi^ (ROI.01) and CD31^low^EMCN^low^ (ROI.02) areas using Leica LAS X software. (**F**) Summary of Figure 4 indicating the occurrence of type H vessels in the induced membrane. Box plots represent the median (line), interquartile range (box), and minimum-to-maximum values (whiskers). *P* < 0.05 was considered statistically significant. BD, bone defect; EMCN, endomucin; IM, induced membrane; Soft, soft tissues. Scale bars: 2,500 μm (**A**), 10 μm (**D**).

Angiogenesis is pivotal in facilitating new bone formation and healing in CSBD (11, 12). Importantly, type H vessels are subtypes of capillaries that contribute to bone development and fracture healing (13, 17). However, it remains unclear whether the microvessels formed in the induced membrane are type H vessels. To substantiate this, 2 critical type H markers, CD31 and EMCN, were detected in the cement-induced membrane of rats, as demonstrated by immunofluorescence (IF) staining. IF results showed increased expression of CD31 (green) and EMCN (red) in the induced membrane compared with the bone defect (BD) and adjacent soft tissues (Figure 5, A–C), and the merged results of CD31 (green) and EMCN (red) in the induced membrane were quantified using Leica Application Suite X (LAS X) software and ImageJ (Supplemental Figure 3A). In the area of type H vessels, signals of CD31 (green) and EMCN (red) highly overlapped compared with CD31-expressing areas (Figure 5, D and E; ROI.01 in the upper panel). This merge was not observed in the corresponding soft tissues (Figure 5D; the lower panels). Our data strongly corroborate the occurrence of type H vessels in the induced membrane (Figure 5F).

To further determine whether the IMT procedure is closely associated regulating purine metabolism and type H vessel formation, femurs of rats were collected to assess type H markers as well as ADSS1 and IMPDH2 at the defect sites (IMT rats: PMMA cement; BD rats: empty defect) and in cortical bone (Supplemental Figure 3B). Consistently, type H vessel formation was increased in IMT rats within the induced membrane and the adjacent bone tissue connected to it, but not at the corresponding sites in BD rats or in cortical bone (Supplemental Figure 3, C–F). ADSS1 and IMPDH2 expression exhibited a similar IMT-versus-BD pattern (Supplemental Figure 4). In addition, quantitative real-time RT-PCR confirmed concordant changes at the mRNA level relative to protein expression (Supplemental Figure 5). Together, these data support that the IMT procedure is associated with increased type H–related neovascularization.

### Effect of purine metabolism on the induction of type H phenotype in HUVECs.

**Figure 6:**
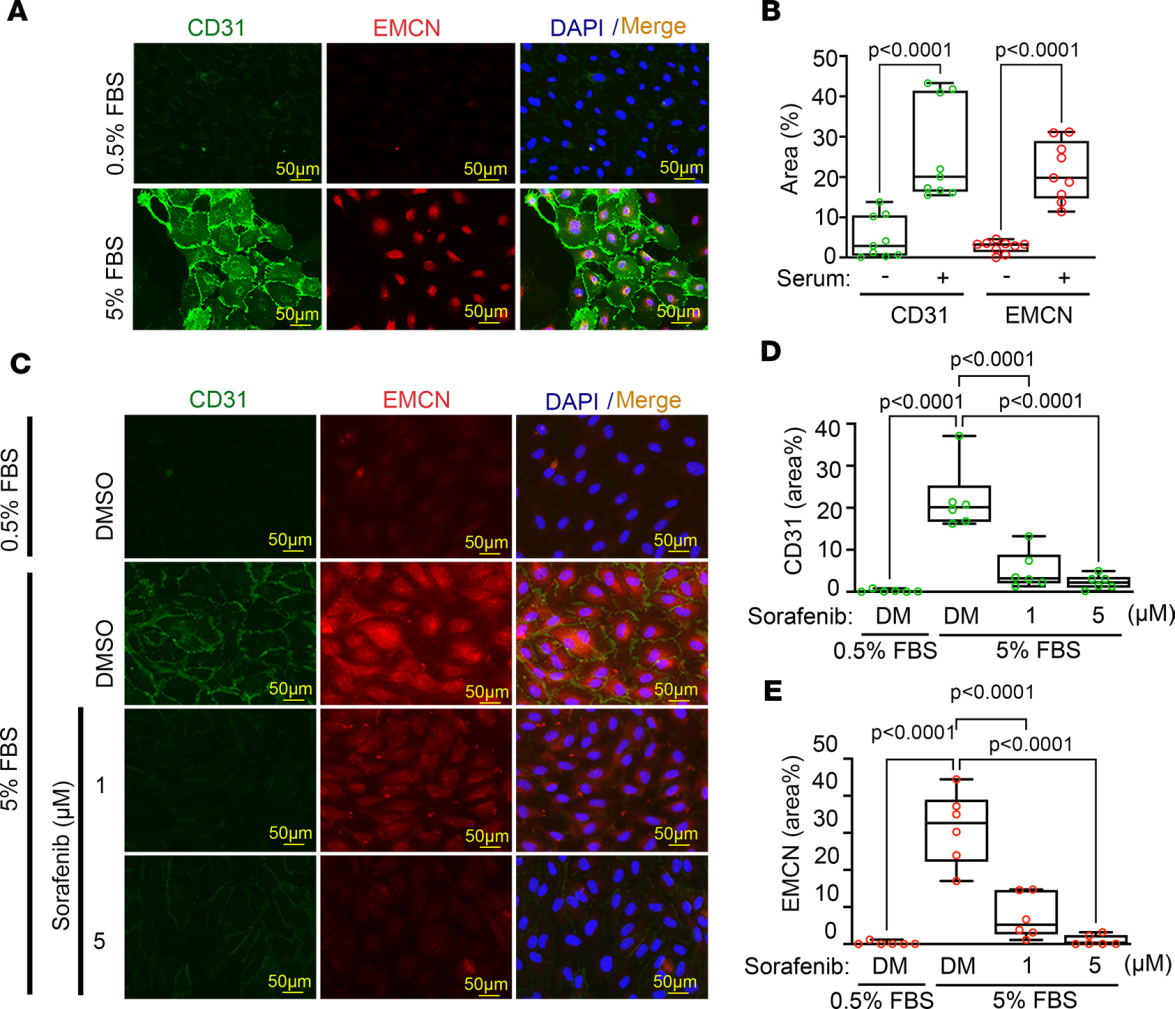
Endothelial activation in HUVECs is associated with changes in type H marker expression. (**A**) HUVECs starved in 0.5% FBS medium for 24 hours were kept in 0.5% FBS (quiescent) or switched to 5% FBS for 3 days (activated), followed by staining with type H markers. All data are presented as box plots. (**B**) In vitro data are based on 3 independent experiments performed in triplicate. Fluorescence intensity was quantified using ImageJ (green: CD31; red: EMCN). (**C**–**E**) HUVECs cultured in 5% FBS (activated) versus 0.5% FBS (quiescent) were treated with sorafenib (Sora) to suppress endothelial activation. Dimethyl sulfoxide (DMSO) was used as solvent control. In vitro data are based on 3 independent experiments performed in duplicate. Fluorescence intensity was quantified using ImageJ (green, CD31; red, EMCN). All data are presented as box plots. Box plots represent the median (line), interquartile range (box), and minimum-to-maximum values (whiskers). *P* < 0.05 was considered statistically significant. Scale bars: 50 μm. BD, bone defect; DM, DMSO; EMCN, endomucin; FBS: fetal bovine serum.

**Figure 7:**
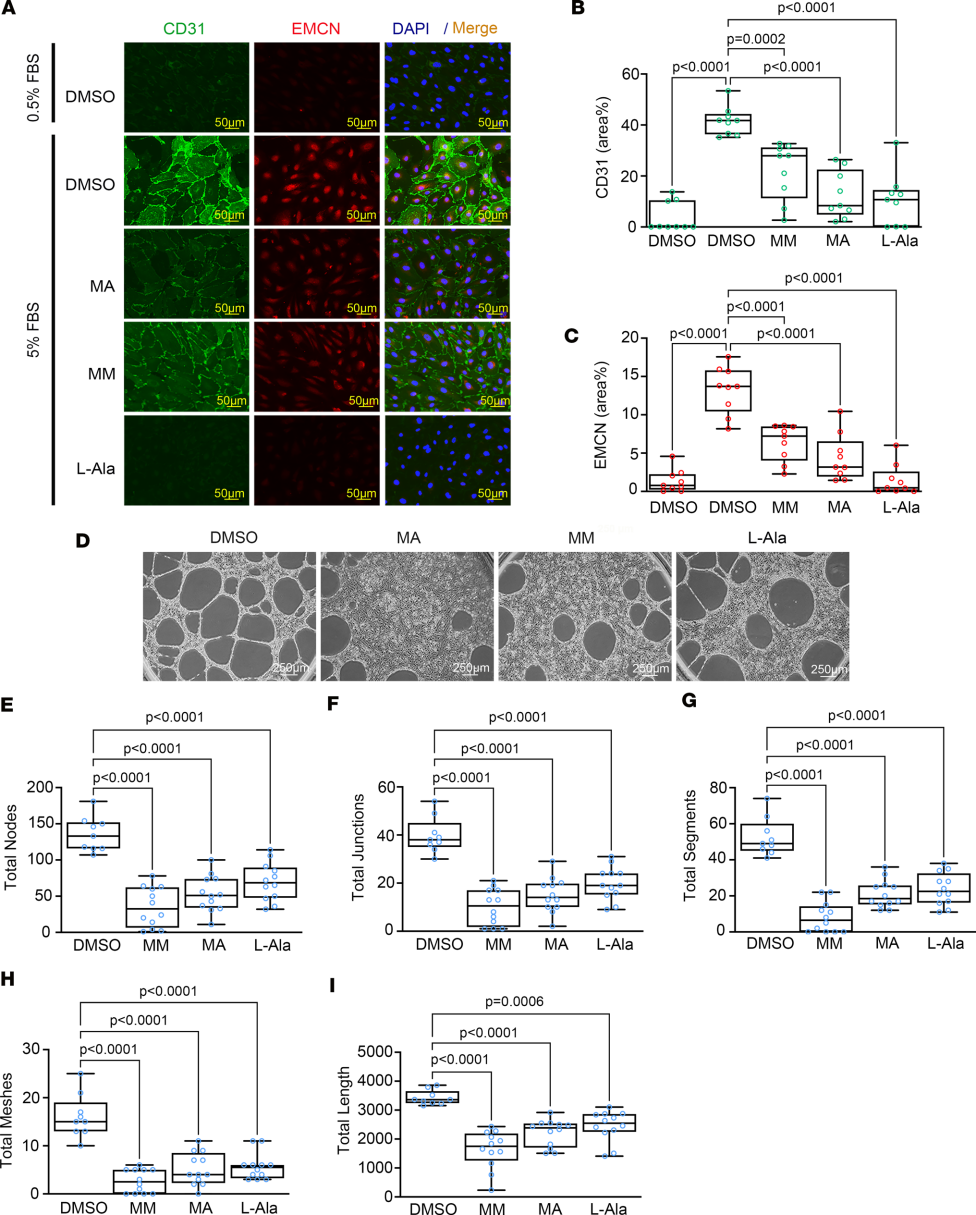
Inhibition of purine metabolism suppresses angiogenic capacity and type H phenotype in HUVECs. HUVECs were seeded into chamber slides with 5% FBS medium, then serum starved in fresh 0.5% FBS medium for 24 hours and treated with purine metabolism inhibitors: mycophenolic acid (MA, 20 μM), mycophenolate mofetil (MM, 20 μM), and L-alanosine (L-Ala, 20 μM). DMSO was used as solvent control. (**A**) Immunofluorescence images of HUVECs after 3-day treatment, stained for DAPI (blue), CD31 (green), and EMCN (red). (**B** and **C**) In vitro data are based on 3 independent experiments performed in triplicate. Fluorescence intensity was quantified using ImageJ (green, CD31; red, EMCN). (**D**) Representative images of tube formation after 24-hour treatment. (**E**–**I**) Quantification of angiogenesis parameters — including total number of nodes, junctions, segments, meshes, and length — was analyzed by ImageJ. In vitro data are based on 3 independent experiments with triplicate measurements. All data are presented as box plots. Box plots represent the median (line), interquartile range (box), and minimum-to-maximum values (whiskers). *P* < 0.05 was considered statistically significant. Scale bars: 50 μm (**A**); 250 μm (**D**). EMCN, endomucin.

Endothelial activation is required for angiogenesis, including that of bone-associated type H vessels (14, 25). To further identify the source of purine metabolites, we collected conditioned media from HUVECs cultured in 0.5% FBS (quiescent) or 5% FBS (activated) and performed untargeted metabolomics. Consistent changes in purine metabolites were observed, including decreased inosine and increased hypoxanthine in the 5% FBS condition compared with 0.5% FBS (Supplemental Figure 6). Two purine enzymes, ADSS1 and IMPDH2, were shown increased in the induced membranes (Supplemental Figure 7, A–F) and expressed in proximity to CD31-positive vascular structures, but no colocalization (Supplemental Figure 7G). Further, immunofluorescent staining of HUVEC demonstrated that ADSS1 and IMPDH2 were indeed expressed in HUVECs and upregulated under endothelial activation (Supplemental Figure 8). These results support an association between purine metabolism and endothelial activation in angiogenesis. FBS-induced activation of HUVECs led to increased CD31 and EMCN expression relative to the resting state maintained in low-FBS medium (Figure 6, A and B). Inhibition of endothelial activation by sorafenib suppressed the type H phenotype in HUVECs, as demonstrated by the decreased expression of CD31 and EMCN (Figure 6, C–E). These data suggest that HUVECs have the potential to exhibit a type H phenotype, which can be linked to the alteration of purine metabolism. Next, the impact of purine metabolism on the type H phenotype was examined by employing inhibitors targeting enzymes associated with the purine metabolites identified in our metabolomic profile. Specifically, purine metabolism was inhibited by targeting IMPDH2 with either mycophenolic acid (MA) or mycophenolate mofetil (MM), as well as ADSS1 with L-alanine (L-Ala). IF staining of HUVECs treated with these inhibitors demonstrated that inhibition of purine metabolism reduced both CD31 and EMCN expression, indicating a loss of the type H phenotype (Figure 7, A–C). In the tube formation assay, treatment with these inhibitors, including MA, MM, and L-Ala, significantly restrained the angiogenic capacity of HUVECs (Figure 7D), as quantified by several parameters, including nodes, junctions, segments, meshes, and lengths (Figure 7, E–I). Notably, this suppressive angiogenesis in HUVECs was accompanied by the downregulation of CD31-EMCN, implying the loss of the type H phenotype under inhibition of purine metabolism. Therefore, the role for purine metabolism in the regulation of type H markers was determined in HUVECs.

### Role of inosine, hypoxanthine, and xanthosine in promoting type H phenotype in HUVECs.

**Figure 8:**
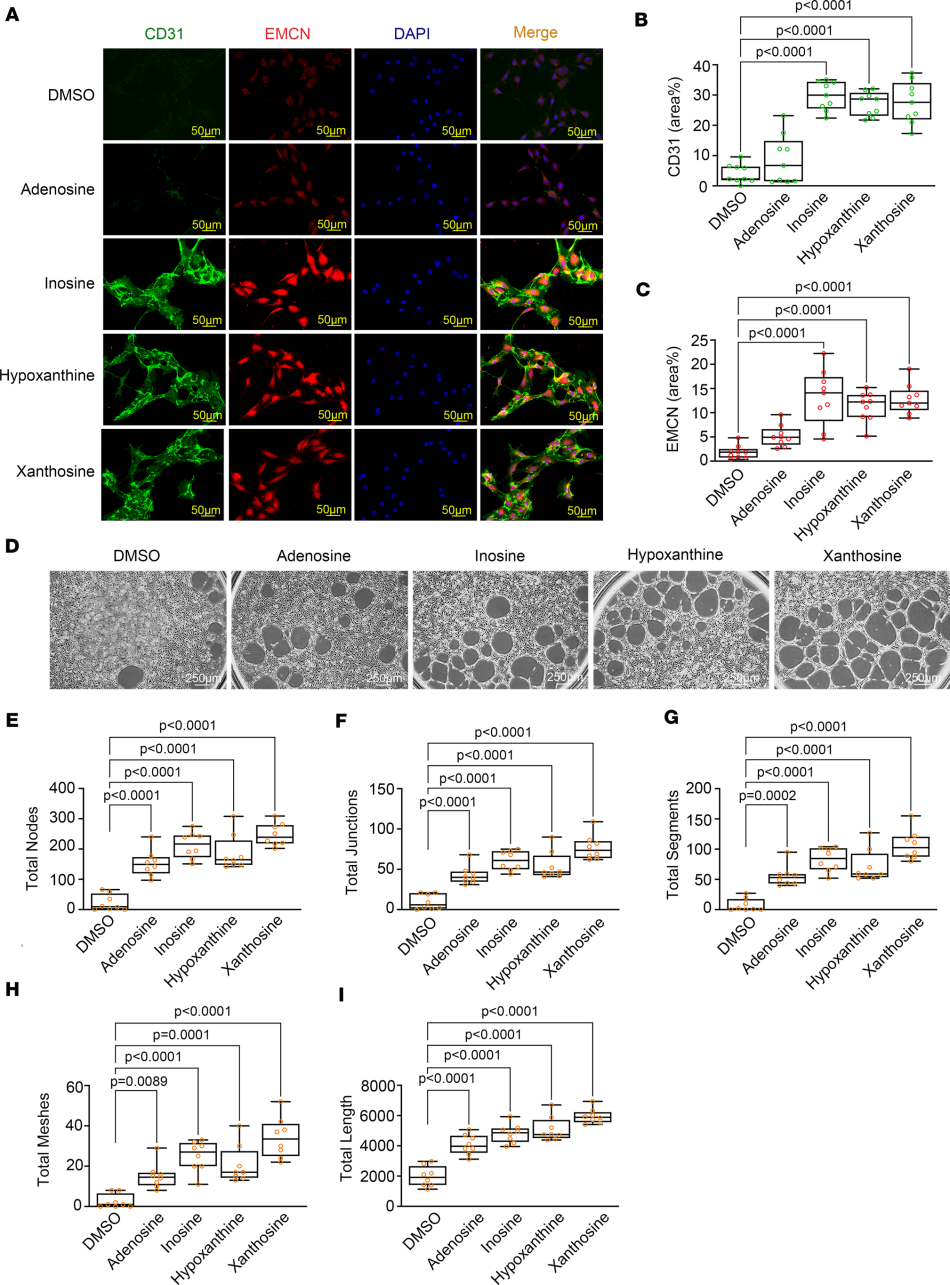
Purine metabolites promote angiogenic capacity and type H phenotype in HUVECs. HUVECs were seeded into chamber slides with 5% FBS medium, then serum starved in fresh 0.5% FBS medium for 24 hours and treated with purine metabolites: adenosine, inosine, hypoxanthine, or xanthosine (each at a concentration of 300 μM). DMSO was used as solvent control. (**A**) Immunofluorescence staining after 3-day treatment, showing DAPI (blue), CD31 (green), and EMCN (red). (**B** and **C**) In vitro data are based on 3 independent experiments performed in triplicate. Fluorescence intensity was quantified using ImageJ (green, CD31; red, EMCN). (**D**) Representative images of tube formation after 8-hour treatment. (**E**–**I**) Quantification of angiogenic parameters, including total number of nodes, junctions, segments, meshes and length, was performed using ImageJ. In vitro data are based on 3 independent experiments with triplicate measurements. All data are presented as box plots. Box plots represent the median (line), interquartile range (box), and minimum-to-maximum values (whiskers). *P* < 0.05 was considered statistically significant. Scale bars: 50 μm (**A**); 250 μm (**D**). EMCN, endomucin.

Another issue to be addressed is whether the metabolites identified from purine metabolism could modulate the type H phenotype in endothelial cells. HUVECs were treated with adenosine, inosine, hypoxanthine, and xanthosine to examine their regulation of the type H phenotype and capacity for angiogenesis. Among the 4 metabolites, inosine, hypoxanthine, and xanthosine promoted the expression of CD31 and EMCN, indicating that these 3 metabolites are essential regulators of the type H phenotype (Figure 8, A–C). Further, tube formation demonstrated that all identified purine metabolites promoted in vitro angiogenesis in HUVECs (Figure 8, D–I). These results collectively corroborate that purine metabolism is highly correlated with the regulation of the type H phenotype, suggesting that these purine metabolites are an imperative inducer of type H vessels in induced membranes.

### Inosine and hypoxanthine as 2 potential serum indicators for local dynamics of induced membrane and type H vessels.

**Figure 9:**
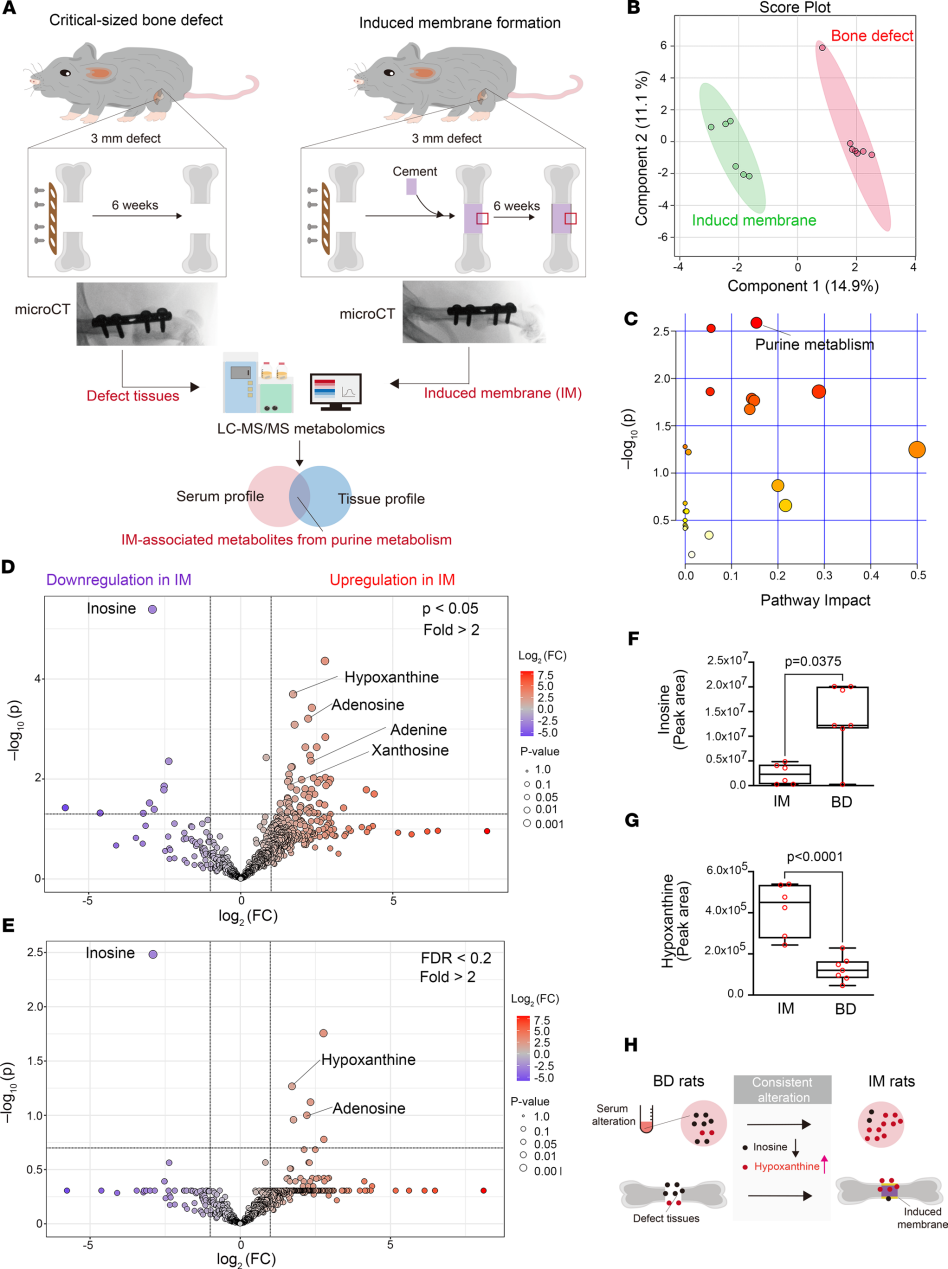
Identification of induced membrane-associated metabolites from purine metabolism. (**A**) Schematic overview of the approach to identify purine metabolites associated with induced membrane (IM). Significant metabolites were defined by criteria of *P* < 0.05, FDR < 0.2, and Fold > 2. (**B**) PLS-DA score plot of tissue samples from IMT (induced membrane, *n* = 6) and BD (bone defect, *n* = 7) rats at week 6. Each data point represents an individual rat. (**C**) Pathway enrichment analysis of tissue metabolomes performed via MetaboAnalyst (hypergeometric test), with pathways ranked by adjusted *P* values (color gradient from white to red on *y* axis) and pathway impact score (*x* axis), where circle size represents ratio of identified metabolites. (**D**) Volcano plot (Student’s *t* test) of tissue metabolites before Benjamini-Hochberg correction. (**E**) Volcano plot (Student’s *t* test) after Benjamini-Hochberg correction. (**F** and **G**) Quantitative profiles of inosine (**G**) and hypoxanthine (**H**) in IM and BD tissue samples. Each dot represents one rat (IM, *n* = 6; BD, *n* = 7) from cohort of IMT as shown in Figure 1 and Figure 2, A–F. Each data point represents an individual rat, and all data are presented as box plots. Box plots represent the median (line), interquartile range (box), and minimum-to-maximum values (whiskers). *P* < 0.05 was considered statistically significant. (**H**) Summary of Figure 7 showing consistent trends of inosine and hypoxanthine between serum (systemic) and induced membrane (local). BD, bone defect; IM, induced membrane; BD, bone defect; IM, induced membrane.

**Figure 10:**
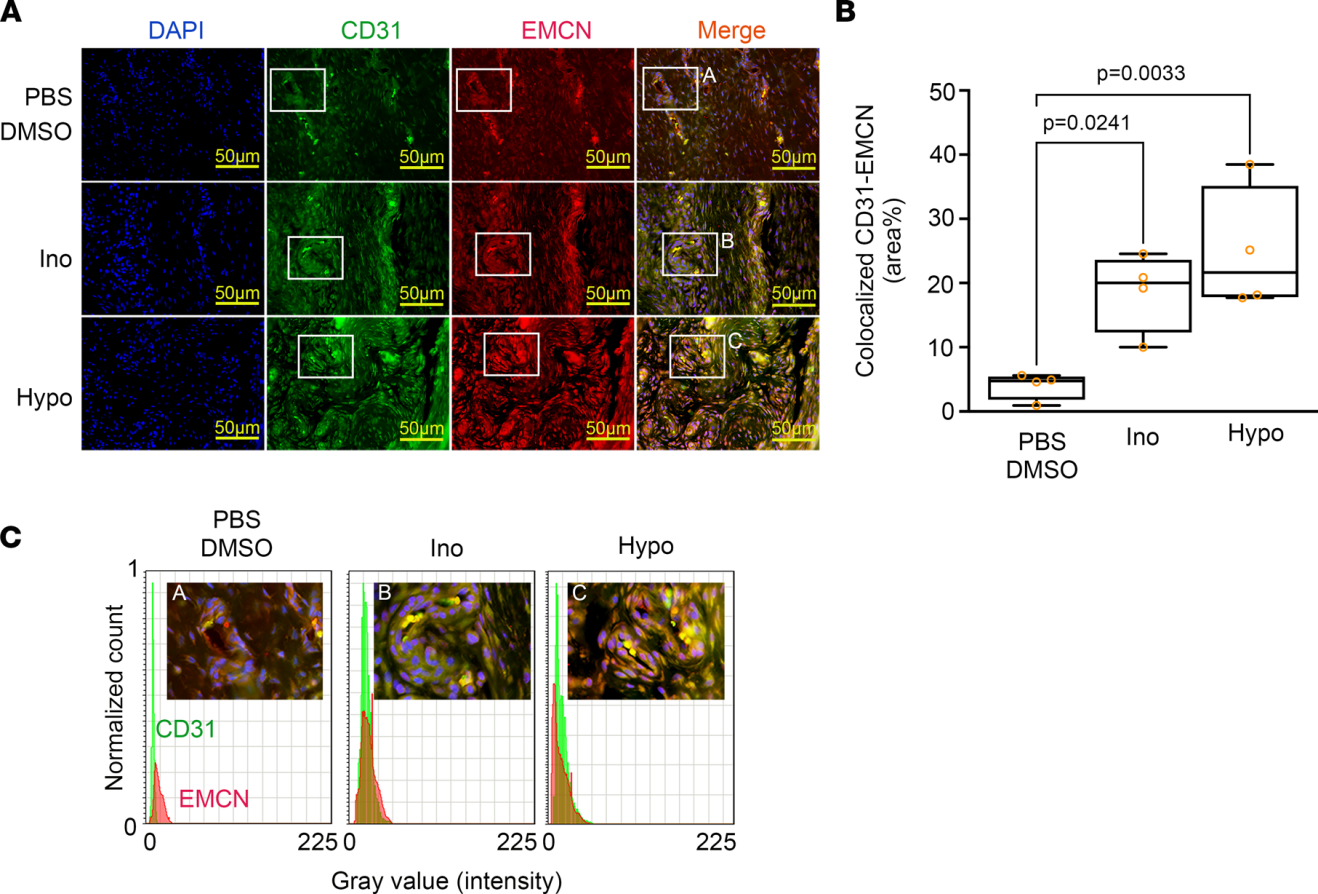
Inosine and hypoxanthine promote the formation of type H vessels in induced membranes. The IMT rats underwent surgical introduction of femur defects with a size of 3 mm, followed by the placement of cement into defect sites for 6 weeks. These rats were administrated with PBS/DMSO (*n* = 4), inosine (200 mg/kg; *n* = 4) and hypoxanthine (20 mg/kg; *n* = 4) 3 times a week. Identical volumes of PBS and 1% of DMSO were mixed together and served as the solvent control. (**A**) The induced membrane was collected to detect expressions of type H markers. (**B**) The colocalization of CD31 and EMCN in induced membranes was determined by using colocalization plugin of ImageJ. Data are presented as area fraction and normalized to DAPI areas. Each dot represents one rat. Box plots represent the median (line), interquartile range (box), and minimum-to-maximum values (whiskers). *P* < 0.05 was considered statistically significant. (**C**) Quantification of CD31^hi^EMCN^hi^ areas, indicated by the white boxes in the figure (ROI.A: PBS/DMSO; ROI.B: Ino; ROI.C: Hypo) using Leica LAS X software. Scale bars: 50 μm. BD, bone defect; EMCN, endomucin; Hypo, hypoxanthine; IM, induced membrane; Ino, inosine.

Fracture healing is considered a localized response, and there is currently no clear evidence in the literature indicating the potential of blood indicators to reflect local fracture healing changes. To further determine whether the serum purine metabolites identified recapitulated local metabolic alterations during IMT, induced membranes from IMT rats were collected and subjected to metabolomic profiling to determine the IM-associated metabolites (Figure 9A). The metabolomics results of induced membranes indicated that purine metabolism was significantly regulated (Figure 9, B and C; BD: bone defect without cement, *n* = 7; IM: induced membrane, *n* = 6), with consistent identification of adenosine, inosine, hypoxanthine, and xanthosine from purine metabolism (Figure 9D). To mitigate the risk of false positives in metabolomics, the Benjamini-Hochberg method was used to determine the significant targets with FDR < 0.2 (Figure 9E). An identical pattern in the serum and induced membrane was required to substantiate their potential as an effective indicator of both circulation and local changes. Our metabolomic analysis showed that inosine and hypoxanthine were 2 potential targets of type H regulators that met this criterion (Figure 9, F and G). These data strongly identify inosine and hypoxanthine as 2 potential serum indicators of type H vessel induction and induced membrane formation (Figure 9H). Furthermore, IMT rats were administered inosine (200 mg/kg) (24) and hypoxanthine (20 mg/kg) (26, 27) intraperitoneally 3 times per week. In the induced membrane, type H vessel signals were increased following inosine and hypoxanthine treatment, as evidenced by enhanced colocalization of type H markers (Figure 10). These data support an association between purine metabolites and type H–related neovascularization within the induced membrane.

## Discussion

In a rat femoral bone defect model with bone cement implantation mimicking IMT, we demonstrated that purine metabolism is closely linked to type H vessel angiogenesis in the induced membrane. Serum inosine and hypoxanthine emerged as potential indicators of IMT, supported by metabolomic profiling that highlighted purine metabolism as the most altered pathway at late stage (week 6). Immunofluorescence demonstrated the occurrence of CD31^+^EMCN^+^ type H vessels in the induced membrane, while HUVEC assays showed inosine and hypoxanthine promoted endothelial activation and the type H endothelial phenotype. Critically, metabolomic analysis of the induced membrane further revealed alterations in inosine and hypoxanthine consistent with the serum analysis, with inosine downregulated and hypoxanthine increased. These findings suggest that circulating purine metabolites may potentially reflect local angiogenic activity, providing potential mechanistic and clinical insights for evaluating IMT outcomes. More importantly, our findings hold potential for clinical application in promoting and accelerating bone healing.

Three primary theories have been proposed to explain the success of IMT: (a) neovascularization within the membrane facilitates osteogenic capacity of the bone graft; (b) the secretion of angiogenic and osteogenic factors that modulate cellular behavior and support new bone formation; and (c) IMT acts as a physical barrier that prevents the infiltration of fibrotic tissue and protects the graft from premature resorption (1, 2, 10). Angiogenesis of new capillaries, resulting from CD31-positive endothelial cells (28), is an indispensable factor involved in induced membrane–mediated healing in IMT (8, 29), transporting abundant angiogenic and osteogenic factors to the defect sites (11, 12). In orthopedic research, studies of IMT have largely addressed the osteogenic function of the induced membrane, with limited attention to angiogenesis. This study, however, investigates the aspect of type H vessel angiogenesis in the induced membrane. In IMT rats, our findings identify purine metabolism as a critical regulator of type H vessel angiogenesis in IMT, offering potential therapeutic strategies for CSBD. Nonetheless, intrinsic differences in bone structure, mechanical loading, and immune responses between rodents and humans may limit direct translation and risk overestimating efficacy. Validation of type H vessel angiogenesis in human samples will therefore be essential to establish clinical relevance and application, thereby addressing this limitation. Consistent with several findings, type H vessels again were shown a type of critical capillaries in new bone formation. Neag et al. indicated that their findings corroborated the importance of type H endothelial cells in promoting trabecular bone formation and patterning by mediating osteoblastic differentiation (16). In a study by Li et al., the endothelial cell–specific deletion of Ybx1 disrupted the morphology of the CD31^hi^EMCN^hi^ endothelium, leading to reduced bone mass, whereas Ybx1 overexpression enhanced angiogenesis-dependent osteogenesis and mitigated bone loss (14). These discoveries provide mechanistic insight into how the induced membrane may contribute to graft integration and regeneration, not only through the secretion of growth factors, but also via active metabolic and angiogenic support.

Type H vessels have high osteogenic potential as a subtype of bone capillaries. Recent investigations have indicated that 2 specific subtypes of the endothelium, type H and type L, are found in bones with relatively high (H) and low (L) expression of CD31 and EMCN (30). CD31^Hi^ EMCN^Hi^ type H vessels possess a columnar morphology and are located in the metaphysis and the periosteum and endosteum of the diaphysis (30). In addition to CD31, this EMCN^Hi^ specialized vessel has been reported to serve as a crucial connection between osteogenesis and angiogenesis (31, 32), termed an angiogenic-osteogenic coupling. It therefore suggests an integrated role for type H vessels in induced membrane–mediated bone formation (13, 30). In contrast, type L vessels are mainly found in the diaphysis and contribute primarily to marrow homeostasis and nutrient transport because of their larger size and slower blood flow than type H vessels (13, 17, 30). Abundant osteoprogenitor cells were shown to surround the type H vessels, rather than the bone marrow–associated type L subtype, leading to rich angiogenic and osteogenic factors (30). Thus, compared with the type H subtype, type L is limited in new bone formation. The high presentation of CD31^Hi^ EMCN^Hi^ type H vessels in our animal model directly confirmed the angiogenic-osteogenic coupling mechanism, leading to the success of IMT.

Metabolomics has emerged as a robust and comprehensive approach to identify functionally relevant metabolites and elucidate their roles in biological functions as well as diseases progressions. Furthermore, metabolic regulation is tightly associated with angiogenesis, osteogenesis, and fracture healing. Notably, fracture healing is characterized by enhanced glycolytic activity, which fuels downstream pathways such as the tricarboxylic acid (TCA) cycle and the pentose phosphate pathway (PPP) (33–35). Notably, our metabolomics results showed that purine metabolism, a downstream pathway of the PPP for nucleotide synthesis, is highly regulated in the induced membrane of IMT rats. According to our findings, the elevated expression of 2 key purine metabolism enzymes, ADSS1 and IMPDH2, corroborated the finding that purine metabolic activity was enhanced in the induced membranes. Furthermore, the inhibition of purine metabolism in HUVECs suppressed the type H phenotype, as evidenced by the reduced expression of CD31 and EMCN. These findings strongly suggest that purine metabolism may serve as a critical target in regulating the formation of the induced membrane.

Inosine and hypoxanthine were identified as key purine metabolites in IMT rats, serving as potential indicators for predicting membrane formation in the current study. Nevertheless, inosine displayed opposite trends in metabolomics of serum and induced membranes compared with neovascularization in membrane, yet cell-based and in vivo experiments using direct treatments confirmed that both metabolites were capable of promoting endothelial activation and the type H phenotype. Our previous study has reported a marked reduction of circulating inosine during the early phase of fracture healing in mice (24), consistent with our present findings in IMT rats. Given that inosine can bind to adenosine receptors and be utilized to activate endothelial cells and induce the type H phenotype, we speculate that its reduction may reflect rapid consumption during fracture healing, merely exceeding the rate of replenishment. Decreases in metabolites can often be associated with beneficial outcomes. For example, the substantial consumption of glutamine following exercise indicates enhanced muscle protein synthesis, contributing to tissue repair and improved adaptive responses (36). Based on our current results, these findings suggest that alterations in purine metabolic pathways may contribute to this phenomenon. Specifically, decreased inosine is linked to elevated hypoxanthine, which may contribute to a type H–like endothelial phenotype and type H vessel–associated neovascularization. Collectively, we propose that a decline in inosine, or altered inosine kinetics, is associated with type H vessel formation. Thus, we suggest that accelerated utilization together with metabolic pathway shifts may underlie this paradoxical pattern. In addition to inosine and hypoxanthine, uric acid may be a potential target as well. In muscle atrophy, muscle cells can release hypoxanthine and xanthine, which are taken up by other cells to produce uric acid (37). Despite negative identification of uric acid in the untargeted metabolomics, targeted method may be an option to extend our finding.

In purine metabolism, adenosine is deaminated by adenosine deaminase (ADA) to form inosine, which is subsequently converted to hypoxanthine through the catalytic action of purine nucleoside phosphorylase (PNP) (38). Notably, hypoxanthine can be reversibly converted back to inosine via a reaction with phosphoribosyl pyrophosphate (PRPP) (38). Inosine monophosphate (IMP) serves as another source of inosine and hypoxanthine, generated through dephosphorylation mediated by 5′-nucleotidase (5′NT) (38). Additionally, XMP can be metabolized to xanthosine through the intermediate xanthosine monophosphate (XMP) (38). Collectively, these data underscore the tightly interconnected mechanisms of adenosine, inosine, hypoxanthine, and xanthosine in purine metabolism. In clinical applications, purine metabolites, including adenosine, inosine, and hypoxanthine, have been implicated in neuroprotective, cardioprotective, anticancer, and antidepressive effects (38). The combined use of inosine, adenosine, and hypoxanthine was associated with reduced depressive symptoms in children diagnosed with major depressive disorder, compared with healthy controls (39). Veres et al. reported that inosine could improve endothelial function in myocardial infarction (40). This underscores the beneficial roles of inosine, hypoxanthine, and xanthosine in the angiogenesis of type H vessels in the induced membrane. According to a study by Moore et al. (41), inosine treatment significantly improved the motor function of rhesus monkeys with bony injury in the arms, indicating the potential application of inosine in eliciting new bone formation. Importantly, our study reveals a critical role of purine metabolism in type H vessel formation and suggests that altered profiles of type H indicators, including inosine, hypoxanthine, and xanthosine, may provide a predictive tool for enhancing the surgical outcomes of IMT.

In light of the lack of reliable methods to assess induced membrane formation and maturity, our study identifies purine metabolism–derived regulators of type H vessels as potential indicators for clinical application. Metabolomic analysis revealed consistent regulation of inosine and hypoxanthine in serum and induced membranes, suggesting their utility as systemic indicators of local changes. Although their ability to monitor membrane angiogenesis remains uncertain, ongoing clinical sample collection and targeted metabolomics will be critical to validate these findings and clarify their therapeutic potential in IMT. Moreover, clinically relevant IMT models incorporating metabolic comorbidities such as aging or diabetes may provide conditions in which induced membrane quality varies, including suboptimal membranes with impaired angiogenic and osteogenic potential. Such inadequate membrane formation could serve as essential negative-control conditions to more rigorously evaluate whether inosine and hypoxanthine reflect candidate indicators associated with IMT-induced membrane formation and whether their dynamics are informative of fracture-healing capacity. Further, the stage II operation will also be conducted to address whether induced membranes treated with inosine or hypoxanthine enhance defect healing due to increased type H vessels in IMT. The blockade of inosine or hypoxanthine productions will also be considered to determine the extent of local and systemic contribution to the neovascularization of type H vessels in the induced membrane. Targeted metabolomics of inosine, hypoxanthine, and others, including uric acid, will be validated in human samples or animal models. These will strengthen the current finding of type H vessels as the critical factors for IMT.

Given the strong link between purine metabolism and type H vessel formation, our findings suggest that inosine and hypoxanthine profiles may help predict induced membrane maturation during IMT. Measuring their serum levels could provide a noninvasive means to assess local metabolic activity and guide optimal strategy for vascularization and osteoinductive capacity. Moreover, their role in type H angiogenesis highlights the potential of targeting purine metabolism to enhance membrane maturation and bone repair. Future studies should validate these markers in clinical settings and explore pharmacological modulation to optimize IMT outcomes for CSBD.

## Methods

### Sex as a biological variable.

Male mice were included for this study to align with present research substantiating the high impact of estrogen on fracture healing in postmenopausal women (42).

### Establishment of a critical-sized bone defect rat model.

Specific pathogen-free male Sprague-Dawley rats (8 weeks old) were purchased from BioLASCO Taiwan in collaboration with Charles River Laboratories. Rats were housed in individually ventilated cages (1–2 per cage) under a 12-hour light/dark cycle. The male animals with normal activity and body weight were included, and each rat was treated as an independent experimental unit. Due to the fact that estrogen strongly affects metabolism and fracture healing capacity, this study only includes male rats to minimize confounding effects. Estrogen can also impact inflammation and thus may impact results if females had been used.

For the IMT model with a critical-sized bone defect, 20 male SD rats were used and randomly grouped to reduce confounders in this study for collecting induced membranes and femurs. A 3-mm defect was created in the femurs using a unilateral surgical approach, following established protocols (43). Rats were divided into two groups: the IMT group (*n* = 9; 3 represented in Figure 1 and 6 in Figure 2), in which the defect was filled with cement, and the bone defect (BD) group (*n* = 11 in Figure 1; 4 rats were excluded due to femur instability), in which no cement was applied. 12 IMT rats, no exclusion in this experiment, were further administered inosine (200 mg/kg, *n* = 4) (24) and hypoxanthine (20 mg/kg, *n* = 4) (26, 27) intraperitoneally three times per week to detect type H markers in induced membranes, as compared to the solvent control group (identical volumes of PBS mixed with 1% of DMSO, *n* = 4). The sample size was determined by our previous pilot study following IACUC approve. To collect the induced membrane, the first stage of IMT was performed in rats under general anesthesia with isoflurane (4%–5% induction in a chamber, 1%–3% maintenance by nosecone, oxygen flow 0.5–1 mL/min). As for euthanasia, it was performed using CO_2_ inhalation at a displacement rate of 30%–70% of the chamber volume per minute, followed by confirmation of death. In terminal experiments (week 6), animals were anesthetized with isoflurane (4%–5%) for induction and maintenance until a surgical plane of anesthesia was reached. Deeply anesthetized animals were then euthanized after cardiac puncture by intravenous injection of potassium chloride (1–2 mEq/kg), with death confirmed by cessation of respiration and reflexes. Anesthesia was confirmed by loss of reflex to toe pinch. Rats were placed laterally with the right leg upward on a 37°C heatpad. The right femur was shaved, residual hair removed with alcoholic swabs, and eyes lubricated with ointment. Iodine was applied for aseptic preparation. A skin incision from the greater trochanter to the knee joint exposed the femur. Stainless-steel bone plates and head screws (Lisen Technology) stabilized the defect. A unilateral 3-mm osteotomy defect was created mid-femur with a surgical drill. Muscle and skin were closed with continuous suturing (5–0 ETHICON Coated VICRYLTM). Analgesia was provided perioperatively and postoperatively in accordance with institutional animal care guidelines and ARRIVE recommendations. Ketoprofen (3 mg/kg, subcutaneous) was administered once daily starting preoperatively and continued for 3 days after surgery. Animals were monitored at least twice daily during the first 72 hours and daily thereafter for signs of pain or distress using predefined welfare criteria (e.g., activity, grooming/posture, weight-bearing/lameness, and body weight). Any animal showing persistent signs of pain or distress despite this regimen was managed according to predefined humane endpoints and institutional veterinary guidance. Postoperatively, rats showed only mild mobility impairment. To reduce feeding difficulty, food was placed on the soft floor for easier access. No notable adverse effects occurred. Body weight was recorded weekly, and at 6 weeks post-surgery, micro-CT was performed on the operated limb to analyze the defect. Serum samples were collected weekly for systemic metabolomic analysis. For the identical control group (week 0; the day of the surgical operation) of serum metabolomics, 3 IMT rats and four randomly selected BD rats were included, as shown in Figure 1. In this study, the exclusion criteria included instability of the CSBD in BD rats (*n* = 4; Figure 1) and hemolysis of serum samples (*n* = 3 at week 0 in Figure 1; *n* = 2 at week 0 and *n* = 3 at week 1 in Figure 2). The induced membranes (IMT, *n* = 6; BD, *n* = 7) and femurs (IMT, *n* = 5; BD, *n* = 5) were analyzed. Three rats (IMT=1, BD=2) were excluded due to loss of tissue integrity during processing. were collected and immediately fixed in 10% formaldehyde (Sigma-Aldrich) for immunohistochemical (IHC) analysis. Furthermore, samples of induced membrane frozen at –80°C were also prepared for the metabolome analysis of local alterations as well as real-time qPCR.

### Untargeted analysis of metabolomics.

Blood serum samples were collected from rats and prepared for untargeted metabolomic profiling at the Clinical Metabolomics Core Laboratory of CGMH. Metabolites were extracted using a methanol-based protocol provided by the Core Laboratory, enabling separation of both polar and nonpolar fractions. Metabolomic profiling was performed using a time-of-flight liquid chromatography-mass spectrometry (TOF LC-MS) method on a Waters ACQUITY UPLC I-Class system integrated with an Xevo G2-XS ToF mass spectrometer (Waters). Analyses were conducted in both positive and negative ionization modes to evaluate alignment quality, facilitate metabolite identification, and ensure data reliability using MADAIL software. Statistical analysis was performed using MetaboAnalyst (version 6.0), an R-based platform. Potential metabolites were identified based on a *P*-value < 0.05, an false discovery rate (FDR) < 0.2, and a fold induction exceeding 2.0-fold.

### Immunohistochemistry of formaldehyde-fixed induced membrane.

Induced membrane tissues were embedded in paraffin blocks, and 4-μm sections were prepared for immunofluorescence staining. The primary antibodies used included adenylosuccinate synthase 1 (ADSS1; A6516, rabbit polyclonal antibody, ABclonal), inosine monophosphate dehydrogenase 2 (IMPDH2; A15626, rabbit polyclonal antibody, ABclonal), CD31 conjugated with Alexa Flour 488 (#42777, clone 89C2, Cell Signaling), and endomucin (EMCN) conjugated with Alexa Flour 555 (BS-4808R-A555, rabbit polyclonal antibody, Bioss). Tissue sections were incubated at 60°C for an hour, deparaffinized in xylene, and rehydrated through a progressive dilution ethanol series. To perform IHC staining, citrate buffer (10 mM citrate and 0.05% Tween 20, pH 6.0) was used as the antigen retrieval buffer. Donkey anti-Rabbit IgG (H+L) Highly Cross-Adsorbed Secondary Antibodies, Alexa Fluor 488 (A21206, Invitrogen) and 555 (A31572, Invitrogen), were used to detect ADSS1 and IMPDH2. Image acquisition was performed with an OLYMPUS CKX41 light microscope, followed by quantification using the ImageJ tool.

### Preparation of an in vitro endothelial cell model derived from human umbilical veins.

Human umbilical vein endothelial cells (HUVEC) were sourced from ScienCell Research Laboratories (#8000). According to the manufacturer’s guidelines, HUVECs maintained viability for at least 15 subcultures. HUVEC was maintained for subculture and seeding with a commercial endothelial growth kit of ScienCell Research Laboratories (ECM, #1001). To mimic the resting state, HUVECs were incubated in a starvation medium composed of ECM supplemented with 0.5% fetal bovine serum, antibiotics (1X PS), and endothelial cell growth supplement (ESGS) for 24 hours. Following serum starvation, cells were cultured in fresh medium containing either 0.5% FBS (to sustain a quiescent state) or 5% FBS (to induce proliferation). Subsequently, cells were treated with sorafenib and/or purine metabolites according to the designated experimental conditions.

### In vitro angiogenesis of HUVECs.

Following the manufacturer’s instructions, in vitro angiogenic capacity was evaluated using an angiogenesis kit (ab204726, Abcam). Serum-starved HUVECs (1 × 10^4^ cells per chamber) were seeded on solidified gel within a μ-slide angiogenesis chamber (IB-81506, ibidi). Cells were subsequently treated with purine metabolism inhibitors or purine metabolites. HUVEC images were acquired at 8- and 24-hours post-seeding using an OLYMPUS CKX41 light microscope. Key angiogenesis metrics, including total nodes, junctions, mesh segments, and vessel length, were quantified using an ImageJ plugin developed explicitly for tube formation analysis.

### Immunofluorescence validation of fibrinolytic and type H markers in HUVECs.

Type H characteristics in HUVECs were assessed via immunofluorescence staining for CD31 and EMCN markers. Cells were cultured on 4-well chamber slides (80426, ibidi) to facilitate optimal phase-contrast imaging. When cultures reached approximately 90% confluence, fixation was performed using 4% formaldehyde in PBS for 10 minutes at ambient temperature, followed by PBS washes. Cells were blocked with 1% BSA for 1 hour. For detection of type H markers, cells were incubated overnight at 4°C with CD31 conjugated with Alexa Fluor 488 (#42777, Cell Signaling) and EMCN conjugated with Alexa Fluor 555 (BS-4808R-A555, Bioss). Subsequently, the cells were stained with DAPI and rinsed with PBS thrice. Fluorescent images were acquired using a Leica fluorescence microscope, and stained areas were quantified using ImageJ software.

### Assessment of endothelial type H marker expression in HUVECs by immunoblotting.

UVECs maintained under 0.5% FBS starvation were treated with synthetic metabolites for 72 hours, followed by preparation of cell lysates using RIPA buffer. Protein samples were separated by electrophoretic SDS-PAGE and transferred to PVDF membranes. Immunodetection was performed using CD31 (A19014, Abclonal), EMCN (A14131, Abclonal), and a loading control (Glyceraldehyde-3-phosphate dehydrogenase (GAPDH) (#2118, Cell Signaling). Blots were visualized using the UVP ChemStudio PLUS system (Analytik), and band intensities were analyzed by densitometry with ImageJ software.

### Assessment of purine enzymes and type H marker expression in rats by real-time qPCR.

Total RNA of defect tissues (*n* = 5, 2 were excluded due to low RNA concentration and quality) and induced membranes (*n* = 4, 2 were excluded due to low RNA concentration and quality) was purified using RNeasy Mini Kit (RNeasy Mini, QIAGEN) according to the manufacturer’s instruction. Further, High-Capacity cDNA Reverse Transcription Kit (43-688-14, Applied Biosystems) was employed to produce cDNA from 0.5 μg of total RNA for each sample. As for RT-qPCR, 1 μL of cDNA was used for each reaction of iQ SYBR Green Supermix (1708882, BioRad) with a CFX96 Touch Real-Time PCR Detection System (Biorad). Forward (F; 10 μM) and reverse (R; 10 μM) primes were included to detect CD31 (F: 5′- CACCGTGATACTGAACAGCAA-3′; R: 5′- GTCACAATCCCACCTTCTGTC-3′) (44), EMCN (F: 5′- AAGCACTGACAGAAACATCCA-3′; R: 5′- ACTGTTGGTCGTTCCTTTAGG-3′) (44), ADSS1 (F: 5′-AGGAGCTAAGCCAGCATGTC-3′; R: 5′-CCTCTTGACTCCGCCTGTG-3′) and IMPDH2 (F: 5′-TCAAGCCAAGASCCTCATCGA-3′; R:5′- AGCGACGGGCATACTCAGA-3′) (45).

### Statistics.

All data are presented as box plots and statistical analyses were performed using GraphPad Prism version 9. For box plots, the center line represents the median, the box indicates the interquartile range (IQR; 25th–75th percentile), and the whiskers extend to the minimum and maximum values, unless otherwise specified. Two-tailed Student’s *t* tests were used for pairwise comparisons, while 1-way ANOVA with Tukey’s post hoc test was performed for analyses involving multiple groups, both at a 95% confidence interval. A *P* value of < 0.05 was considered statistically significant. For metabolomic results, multiple comparisons were corrected by the Benjamini-Hochberg method, and PLS-DA was applied for dimensionality reduction and group separation. Based on prior reports (46–48), significance of metabolites was defined by 3 criteria: *P* value < 0.05, FDR < 0.2, and fold change exceeding 1.5 in serum or 2.0 in tissues. The discrepancy in fold thresholds between serum and tissues reflects the greater abundance and complexity of signals in tissue samples.

### Study approval.

The animal study was approved by the IACUC of Chung Gung Memorial Hospital, Linkou (IACUC-2023121501), and conducted in an AAALAC-accredited facility.

### Data availability.

The data are provided in the Supporting Data Values XLS file. The data supporting the findings of this study are also available from the corresponding author upon reasonable request. Please provide a brief description of the proposed use when making a request.

## Author contributions

YHH: Conceptualization, Funding acquisition, writing, review, and editing. GLL: Conceptualization, data curation, supervision, formal analysis, investigation, methodology, project administration, validation, writing – original draft. YCL: Investigation and validation. MFC: Investigation and validation. YC: Investigation and validation. YYW: Data curation, investigation, methodology, validation. CCH: Conceptualization, supervision, Funding acquisition, writing, reviewing, and editing. This study was supported by funding from YHH and CCH. GLL contributed to the organization and supervision of the experiments to ensure their completion. Thus, YHH and GLL were assigned as co-first authors.

## Conflict of interest

The authors have declared that no conflict of interest exists.

## Funding support

- Chang Gung Memorial Hospital of Linkou (CMRPG3P0271, awarded to Chih-Chien Hu).
- Chang Gung Memorial Hospital of Linkou (CMRPG3P0272, awarded to Chih-Chien Hu).
- Chang Gung Memorial Hospital of Linkou (CORPG3M0463, awarded to Chih-Chien Hu).
- Chang Gung Memorial Hospital of Linkou (CMRPG3Q0371, awarded to Yung-Heng Hsu).
- National Science and Technology Council of Taiwan grants (NSTC 113-2314-B-182A-101 awarded to Yung-Heng Hsu).
- National Science and Technology Council of Taiwan grant (NSTC 114-2314-B-182A-071 -MY3, awarded to Yung-Heng Hsu).

## Supplementary Material

Supplemental data

Supporting data values

## Version 1 07/22/2026

Electronic publication
