# Supplementary material for: Purine metabolism enhances neovascularization of type H vessels in the induced membrane technique
Source: JCI Insight. 2026 Jul 22;11(14):e200820. doi: 10.1172/jci.insight.200820 (PMC13461154; doi:10.1172/jci.insight.200820)
Supplement: Supplementary file 1 [file jciinsight-11-200820-s269.pdf]

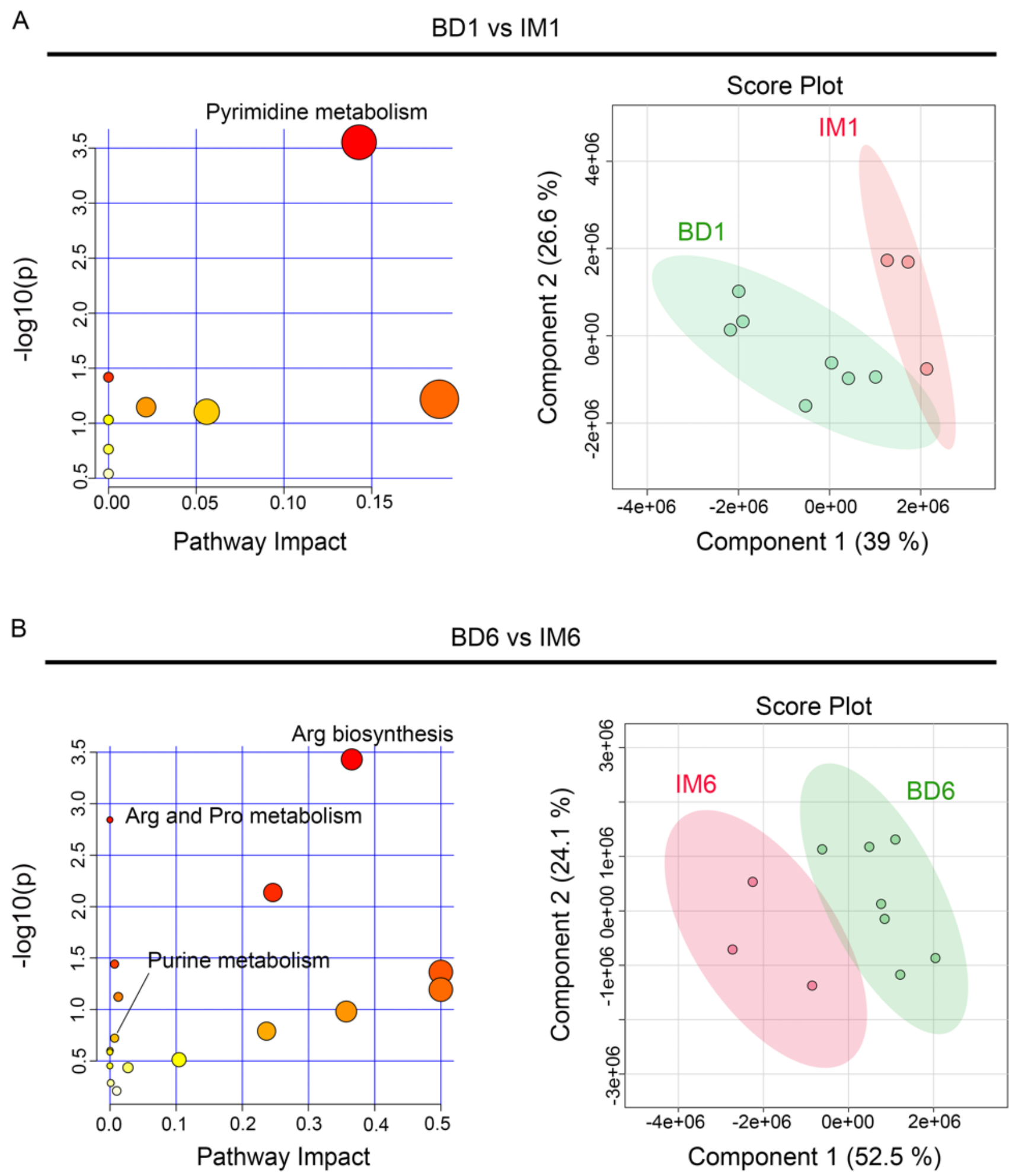

Supplementary Figure 1. The time-dependent analysis of serum metabolomics.

(A-B) Metabolic pathway enrichment analyses and partial least squares discriminant analysis (PLS-DA) of serum samples collected from both bone defect (BD) and induced membrane technique (IMT) rats. Analyses were performed using MetaboAnalyst, which ranked enriched pathways (Hypergeometric Test) based on adjusted p-values (color gradient from white to red on the y-axis) and pathway impact scores (x-axis), with circle size indicating the number of identified metabolites relative to total pathway components. BD: bone defect, IM: induced membrane, 1: week 1, 6: week 6.

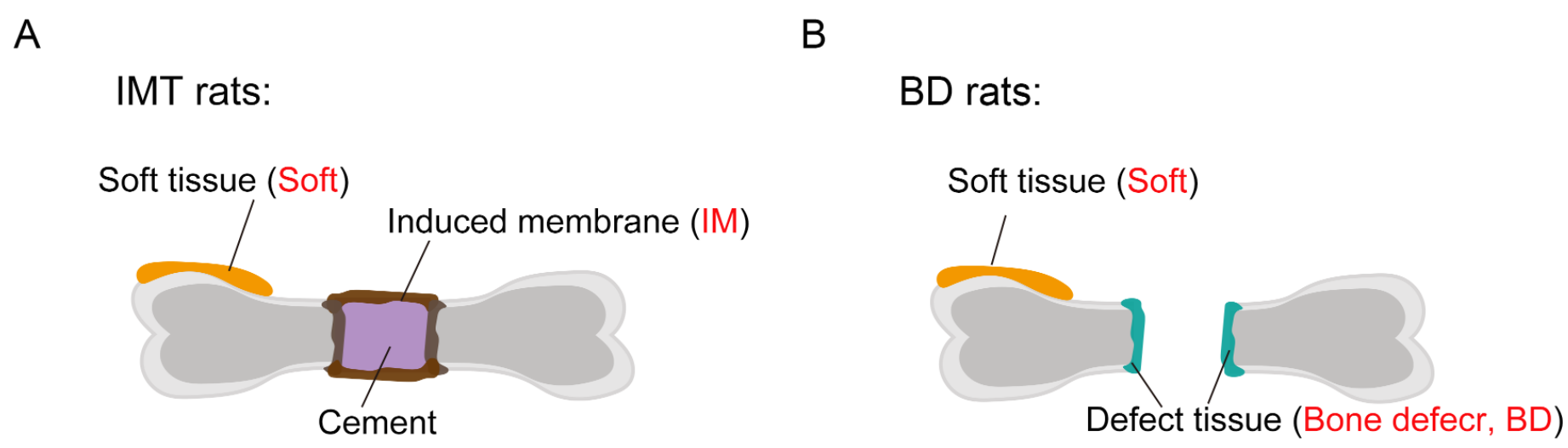

Supplementary Figure 2. Tissue collections from the IMT and BD rats.

(A) IMT (induced membrane technique) rats. (B) BD (bone defect) rats. Soft tissues, as defined as Soft (orange color), were randomly collected from both of IMT and BD rats at the site near joints and far from defects. Induced membranes (IM, brown color) were formed and surrounded on the surface of PMMA cements with a form of thin layer. The relative tissues (defect tissues, green color) from BD rats was defined as Bone defect (BD).

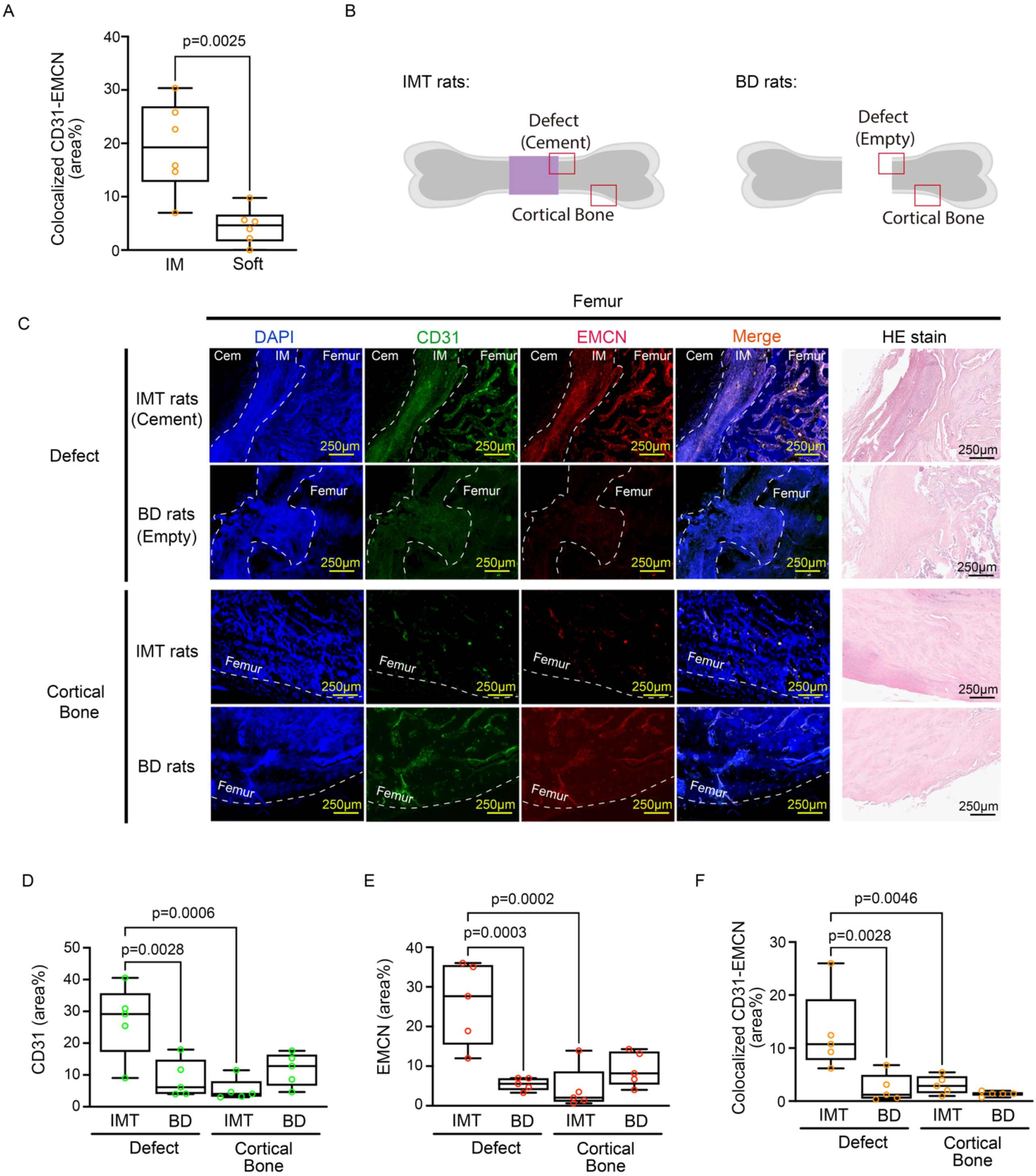

Supplementary Figure 3. Type H vessels is induced by cement near the defect sites of femurs in IMT rats.

Supplementary Figure 3. Type H vessels is induced by cement near the defect sites of femurs in IMT rats.

(A) The colocalization of CD31 and EMCN in induced membrane was determined by using colocalization plugin of ImageJ. (B) Schematic presentation of images captured and defined as defect sites and cortical bones. (C) Representative immunohistochemical images showing type H vessels co-expressing CD31 (green), EMCN (red), with merged orange–yellow signals, in femurs subjected to either bone defect (BD; n=5) or induced membrane technique (IMT; n=5) procedures. The white dashed lines indicated the location of induced membrane (IM) toward cements (Cem) in IMT rats, the corresponding sites (empty: no cement) of BD rats were also shown by the lines. Hematoxylin and Eosin (HE) staining indicated the tissue morphology. (D-E) Quantification of CD31 (D) and EMCN (E) signals using ImageJ. (F) The colocalization of CD31 and EMCN in femurs was determined by using colocalization plugin of ImageJ. Data are presented as area fraction and normalized to DAPI areas. Each dot represents one rat. Box plots represent the median (line), interquartile range (box), and minimum to maximum values (whiskers). $p < 0.05$ was considered statistically significant. BD: bone defect, Cem: cement, EMCN: endomucin, IM: induced membrane, IMT: induced membrane technique.

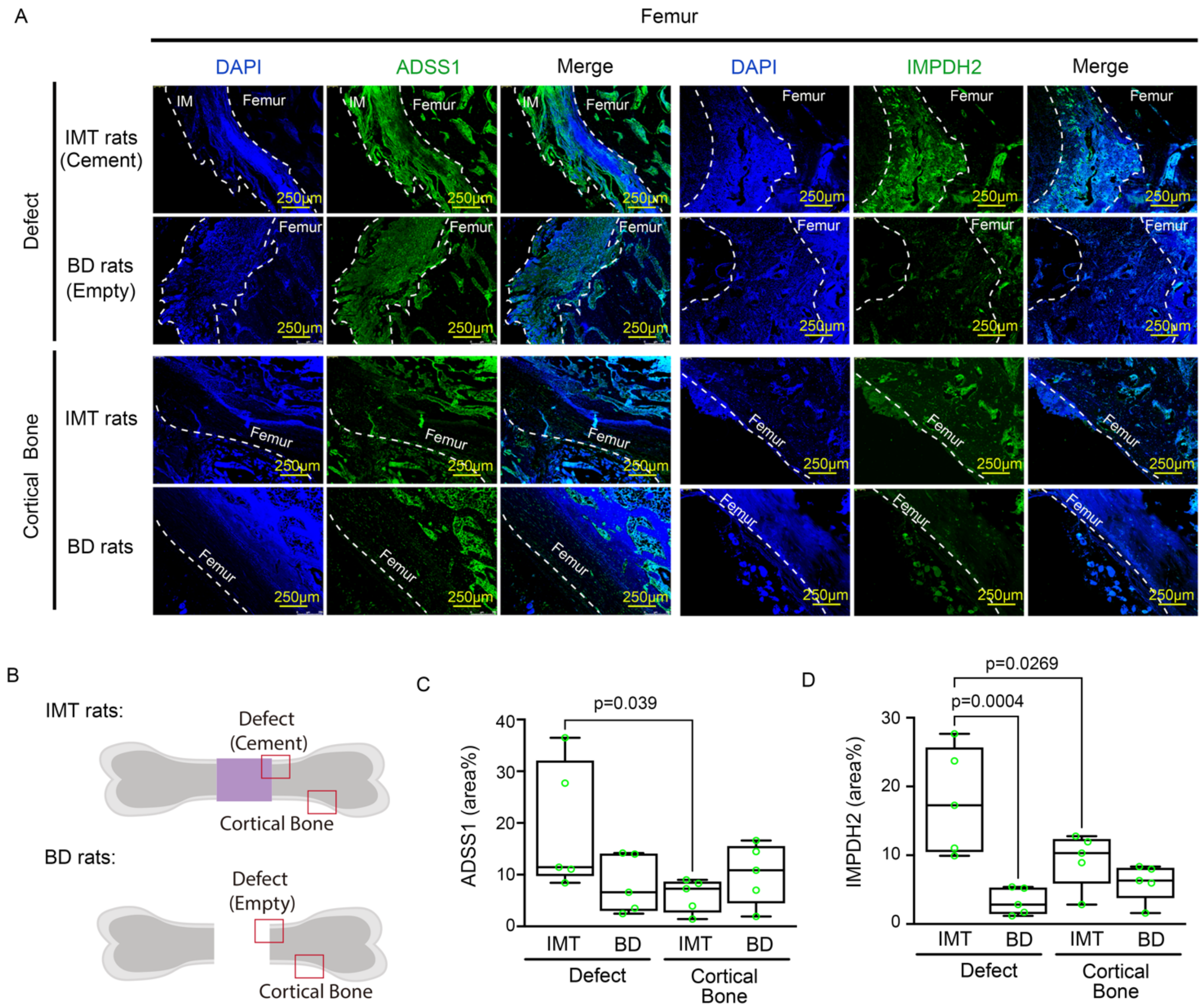

Supplementary Figure 4. The expression of ADSS1 and IMPDH2 are increased near the defect sites of femurs in IMT rats.

Supplementary Figure 4. The expression of ADSS1 and IMPDH2 are increased near the defect sites of femurs in IMT rats.

(A) Representative immunohistochemical images showing expression of ADSS1and IMPDH2 in femurs subjected to the induced membrane technique (IMT; n=5) or bone defect (BD, n=5) procedures. The white dashed lines indicated the location of induced membrane (IM) toward cements (Cem) in IMT rats, the corresponding sites (empty: no cement) of BD rats were also shown by the lines. (B) Schematic presentation of images captured and defined as defect sites and cortical bones. (C-D) Quantification of ADSS1 (C) and IMPDH2 (D) staining using ImageJ. Each dot represents one animal. Data are presented as area fraction and normalized to DAPI areas. Box plots represent the median (line), interquartile range (box), and minimum to maximum values (whiskers). $p < 0.05$ was considered statistically significant. ADSS1: adenylosuccinate synthase 1, BD: bone defect, IMPDH2: inosine monophosphate dehydrogenase 2, IM: induced membrane.

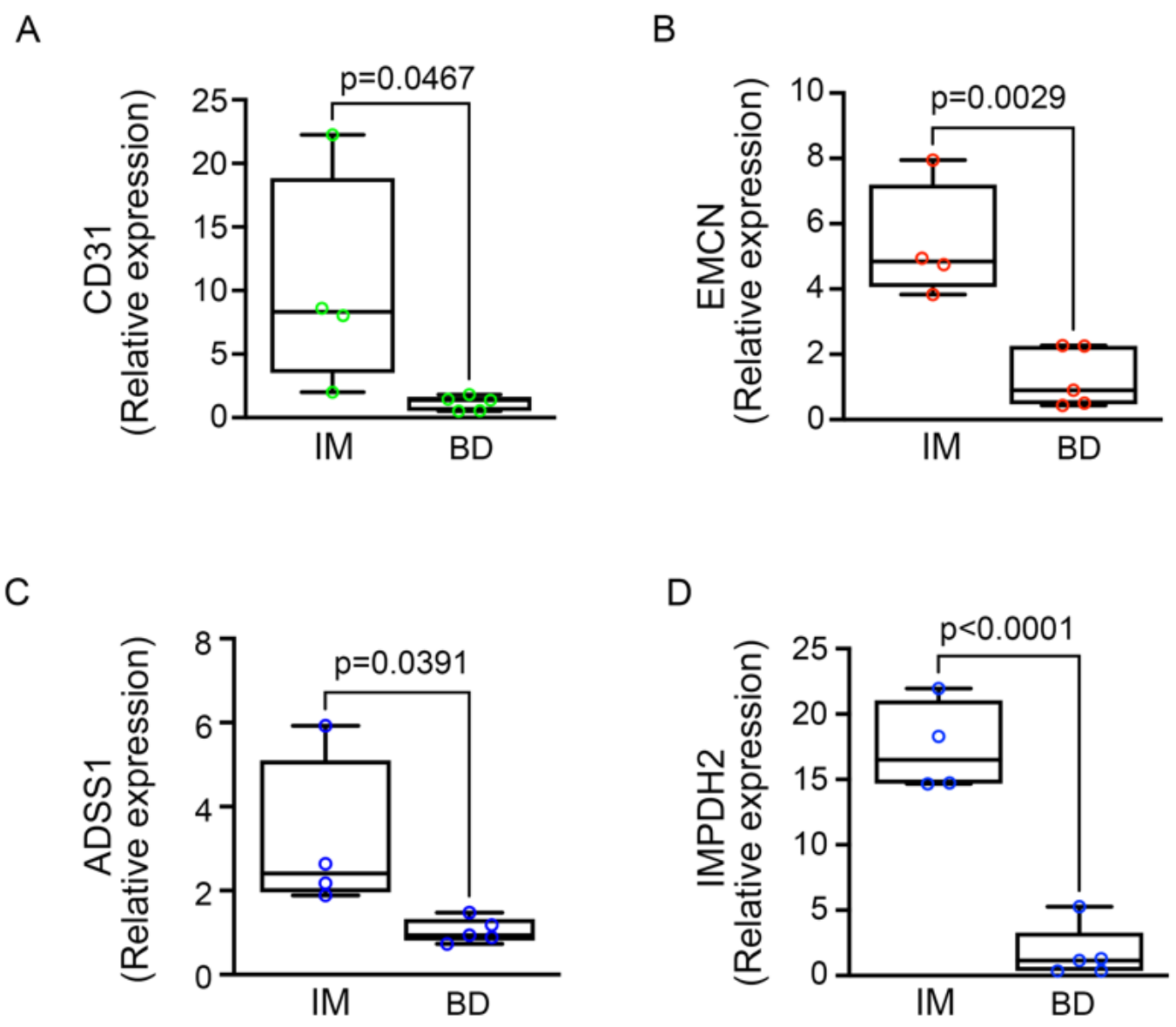

Supplementary Figure 5. The qPCR analysis of CD31 and EMCN in the induced membrane.

Induced membrane tissues from IMT rats (n=4) as well as defect tissues from BD rats (n=5) were collected for quantitative PCR (qPCR) analysis to detect type H markers, including CD31 (A), EMCN (B), ADSS1 (D) and IMPDH2 (D). Data are presented as relative expression to internal control. Each dot represents one rat. Box plots represent the median (line), interquartile range (box), and minimum to maximum values (whiskers). $p < 0.05$ was considered statistically significant. ADSS1: adenylosuccinate synthase 1, BD: bone defect, EMCN: endomucin, IM: induced membrane, IMPDH2: inosine monophosphate dehydrogenase 2.

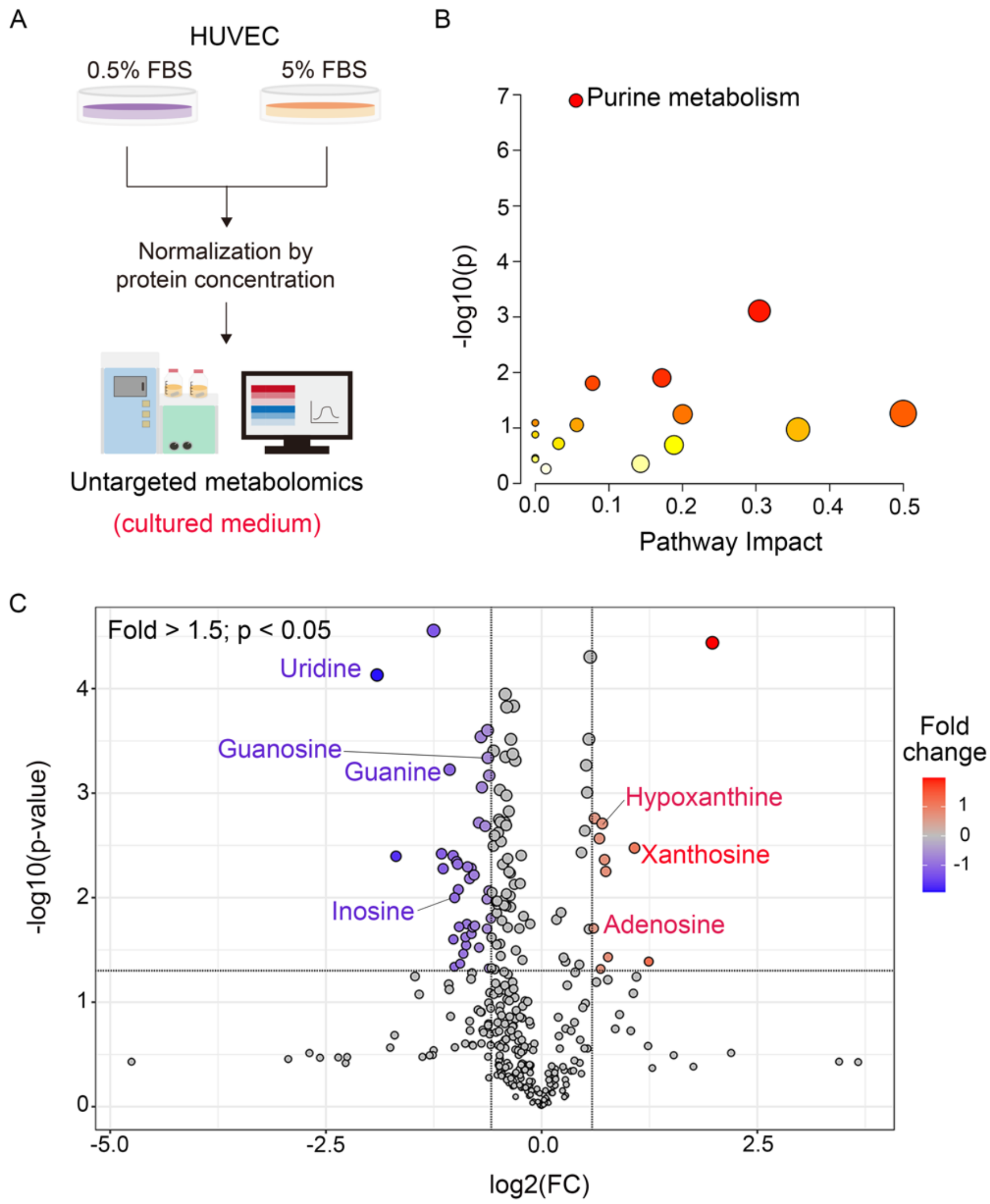

Supplementary Figure 6. The metabolomic analysis of cultured medium from HUVECs.

Supplementary Figure 6. The metabolomic analysis of cultured medium from HUVECs.

(A) Untargeted metabolomic profiling was performed on culture medium collected form HUVECs subjected to either starvation (0.5% FBS, n=3) or growth (5% FBS, n=3) conditions for three days. Metabolites were considered significant if they met all three criteria: $p < 0.05$, FDR < 0.2, and Fold > 1.5. (B) Metabolic pathway enrichment analyses of culture medium collected form HUVECs. Analyses were performed using MetaboAnalyst, which ranked enriched pathways (Hypergeometric Test) based on adjusted p-values (color gradient from white to red on the y-axis) and pathway impact scores (x-axis), with circle size indicating the number of identified metabolites relative to total pathway components. (C) The volcano plot (Student's t-test) of purine metabolism was highlighted. Pathways were considered significant if they met the following criteria: $p < 0.05$. Metabolites were considered significant if they met the following criteria: $p < 0.05$, FDR < 0.2, and Fold>1.5. HUVEC: human umbilical vascular endothelial cell, FBS: fetal bovine serum.

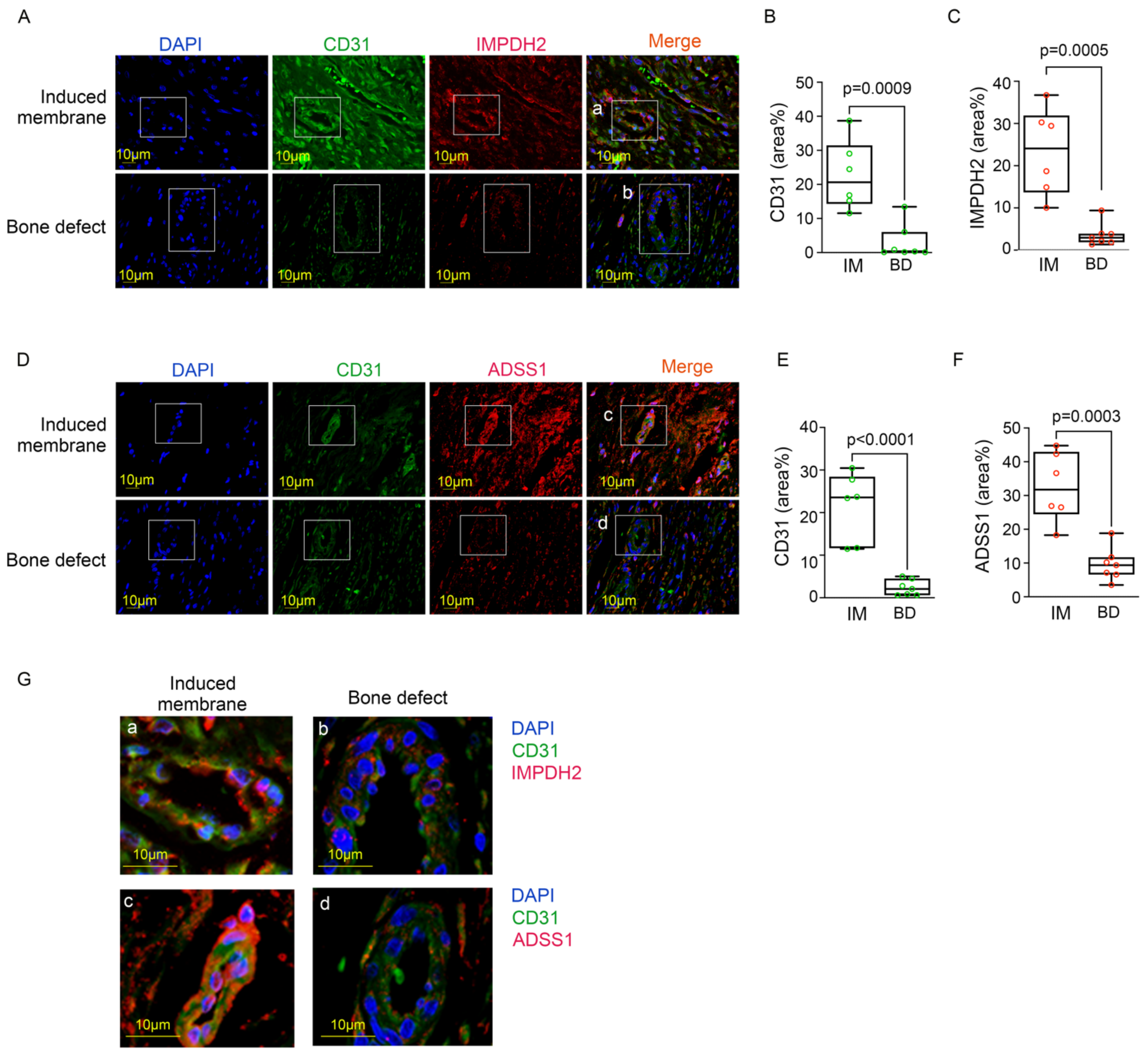

Supplementary Figure 7. The expression of both ADSS1 and IMPDH2 are not colocalized to CD31 in induced membranes.

Supplementary Figure 7. The expression of both ADSS1 and IMPDH2 are not colocalized to CD31 in induced membranes.

(A) Representative immunohistochemical images showing expression of CD31 (green) and IMPDH2 (red) in femurs subjected to the bone defect (BD; n=7) or induced membrane technique (IMT; n=6) procedures. (B-C) Quantification of CD31 (B) and IMPDH2 (C) staining using ImageJ. (D) Representative immunohistochemical images showing expression of CD31 (green) and ASDD1 (red) in femurs subjected to the bone defect (BD; n=7) or induced membrane technique (IMT; n=6) procedures. (E-F) Quantification of CD31 (E) and IMPDH2 (F) staining using ImageJ. In *in vivo* data, each dot represents one animal. Data are presented as area fraction. All data are presented as box plots. Box plots represent the median (line), interquartile range (box), and minimum to maximum values (whiskers). $p < 0.05$ was considered statistically significant. (G) Enlarged figures of IMPDH2 (a-b) and ADSS1 (c-d). ADSS1: adenylosuccinate synthase 1, BD: bone defect, IMPDH2: inosine monophosphate dehydrogenase 2, IM: induced membrane.

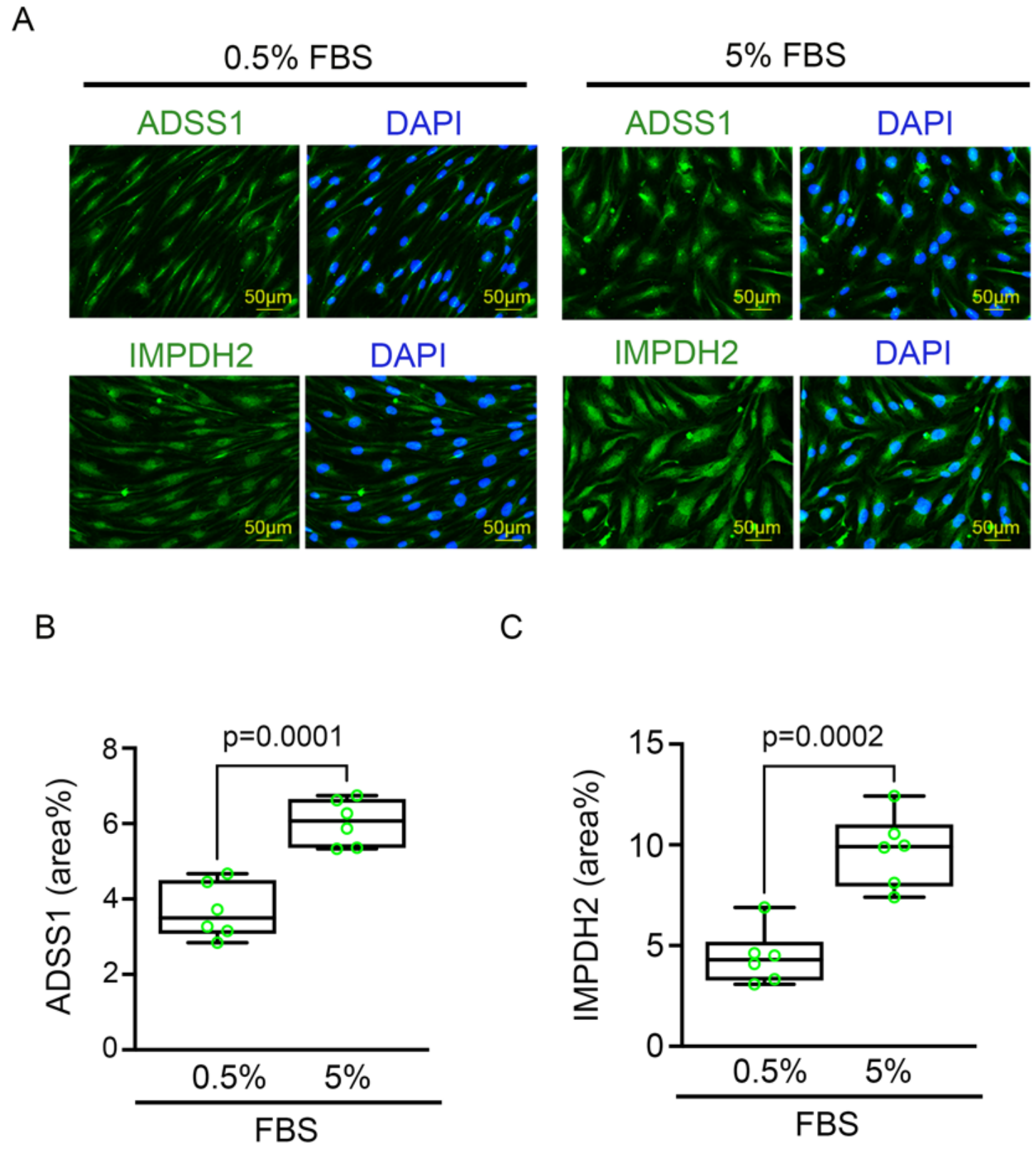

Supplementary Figure 8. ADSS1 and IMPDH2 are expressed and increased in endothelial activation.

HUVECs were seeded into chamber slides with 5% FBS medium, then serum-starved in fresh 0.5% FBS medium for 24 hours and followed by incubation in 5% FBS (activated) versus 0.5% FBS (quiescent) for three days prior immunofluorescent staining. (A) Immunofluorescence image of DAPI (blue), ADSS1 (green, left panel) and IMPDH2 (green, right panel). (B-C) In vitro data are based on two independent experiments performed in triplicate. Fluorescence intensity of ADSS1 (B) and IMPDH2 (C) was quantified using ImageJ. All data are presented as box plots. Box plots represent the median (line), interquartile range (box), and minimum to maximum values (whiskers). $p < 0.05$ was considered statistically significant. ADSS1: adenylosuccinate synthase 1, IMPDH2: inosine monophosphate dehydrogenase 2.
